# Steroidal glycoside profile differences among primary roots system and adventitious roots in *Solanum dulcamara*

**DOI:** 10.1007/s00425-023-04072-9

**Published:** 2023-01-16

**Authors:** Ilaria Chiocchio, Nerea Pérez Andrés, Redouan Adam Anaia, Nicole M. van Dam, Fredd Vergara

**Affiliations:** 1 Molecular Interaction Ecology, German Centre for Integrative Biodiversity Research (iDiv) Halle-Jena-Leipzig, Puschstrasse 4, 04103 Leipzig, Germany; 2 Department of Pharmacy and Biotechnology, University of Bologna, Via Irnerio 42, 40126 Bologna, Italy; 3 Institute of Biodiversity, Friedrich Schiller University, Dornburger-Str. 159, 07743 Jena, Germany; 4 Leibniz Institute of Vegetable and Ornamental Crops (IGZ), Theodor-Echtermeyer-Weg 1, 14979 Großbeeren, Germany

**Keywords:** Chemodiversity, Glycosides, LC–MS, Mass spectrometry, Solanaceae, Steroids

## Abstract

**Main conclusion:**

*Solanum dulcamara* primary and adventitious roots showed qualitative and quantitative differences in their steroidal glycosides profile. This opened new venues to evaluate the bioactivity of these molecules in belowground ecosystems.

**Abstract:**

*The Solanum* genus is characterized by the presence of steroidal glycosides (SGs) that confer herbivore resistance and serve as drug precursors in the pharmaceutical industry. *Solanum dulcamara* is a self-compatible, sexually reproducing species that produces seeds after buzz-pollination. In addition, primordia on the stem facilitate clonal propagation via adventitious root (AR) formation. ARs contain aerenchyma being developmentally and morphologically different from primary roots (PRs). Therefore, we hypothesized that ARs and PRs have different SG profiles. Aiming to assess differences in SGs profiles in *S. dulcamara* roots in relation to their origins and morphologies, we used liquid chromatography coupled to electron spray ionization quadruple time of flight mass spectrometry (LC-ESI-qToF-MS) to profile SGs from PRs and ARs of seven *S*. *dulcamara* individuals. Mass fragmentation pattern analysis indicated the presence of 31 SG-type structures, including those with spirostans and furostans moieties. We assigned the 31 structures to 9 classes of steroidal aglycons (SAgls) that differ in hydroxylation and degree of unsaturation. We found that SAgls were conjugated with di-, tri- and tetra saccharides whereby one compound contained a malonylated sugar. Principle component analysis showed that SG profiles of PRs and ARs separated on the first principal component, supporting our hypothesis. Specifically, PRs contain higher number of SGs than ARs with some compounds exclusively present in PRs. Our results reveal a high level of novel chemodiversity in PRs and ARs of *Solanum dulcamara*. The knowledge gained will deepen our understanding of SGs biosynthesis and their functional role in plant-environment interactions.

**Supplementary Information:**

The online version contains supplementary material available at 10.1007/s00425-023-04072-9.

## Introduction

During land colonization, plants evolved roots to provide physical support and water uptake when growing on land. According to fossil records and the root anatomy of extant vascular plants, rhizoid-based rooting systems in early land plants likely developed from dormant meristems on aerial tissue when in proximity to soil or exposed to flooding (Dawood et al. 2014; Mhimdi and Pérez-Pérez 2020). In extant plants, roots developing from aerial tissues are known as adventitious roots -ARs- (from Latin *adventicius*: foreign). Ontogenetically, ARs have a unique origin. Whereas primary roots (PRs) develop from the embryo’s radicle, ARs develop from adult, non-root vascular tissue (Liu et al. 2018). The PRs include the taproot from which lateral (i.e., secondary) roots grow (Motte et al. 2019). The different origins of PRs and ARs also are reflected by morphological differences.

ARs and lateral roots participate in several, comparable primary functions. They anchor the plant and take up water and nutrients. ARs are expressed constitutively in some species, or upon induction by environmental factors in others (Gonin et al. 2019). The latter type of ARs develops as an adaptive response to various stresses -including wounding and flooding- and are a key component for vegetative propagation (Bellini et al. 2014; Dawood et al. 2014; Alaguero-Cordovilla et al. 2021). Like the aerial parts, roots are involved in a wide range of ecological and environmental interactions governed by plant specialized, or secondary, metabolites (PSMs) (Steffens and Rasmussen 2016). PSMs produced by roots can be exuded, stored in root tissues or they can be distributed to aboveground tissues.

Considering the different origins and morphologies of ARs and PRs, our hypothesis is that these roots also differ in their phytochemical profiles. In addition, ARs and PRs function in different environments. ARs commonly grow closer to, or even over the soil surface, whereas PRs grow deeper into the soil. This means that these two root types are exposed to different biotic and abiotic factors which can, in turn, influence their metabolome (Tsunoda and van Dam 2017).

*Solanum dulcamara* (Solanaceae) is a wild relative of tomato and evolved in Northern Eurasia. It is a morphologically plastic, woody, perennial plant with phenotypes including shrubs and vines (Savinykh and Konovalova 2019; Savinykh and Shabalkina 2020). *S*. *dulcamara* grows in a modular fashion and is found in contrasting environments, from flooded plains to dry dunes (Zhuravlyeva and Savinykh 2014; Calf et al. 2018). In flooded habitats, *S*. *dulcamara* develop ARs, which grow from preformed primordia on the stem (Terras 1897; Dawood et al. 2016; Zhang et al. 2017; Yang et al. 2018).

Chemically, *S*. *dulcamara* is characterized by the production of steroidal glycosides -SGs- which are synthetically derived from cholesterol (Köthe 1980; Lee et al. 1993; Bednarz et al. 2019; Kaunda and Zhang 2019). The corresponding steroidal sapogenins, also known as steroidal aglycones (SAgls), have been reported to occur in its roots (Willuhn and Kun-anake 1970). However, these authors did not specify the type of roots under investigation, which makes it unclear whether SGs are found in PRs as well as in ARs.

Whereas it is known that *S. dulcamara* leaves and fruits display genetically fixed SG chemotypes (Willuhn 1966, 1968), no information is available about the level of chemical diversity in roots. Considering the ecological relevance of SG chemodiversity in aboveground plant–herbivore interactions (Calf et al. 2018, 2019), analyzing ARs and PRs SG profiles is relevant for understanding root chemical ecology.

In addition, the chemodiversity of SGs holds great potential for human applications. In fact, SGs and their SAgls have been reported to display different biological and pharmaceutical activities, including anticancer, antimicrobial, and antipyretic effects (Zhao et al. 2021) and efforts are made to produce them at an industrial scale for pharmaceutical applications (Yang et al. 2002; Bailly 2021; Normandin and Boundreault 2021).

Here, we investigate the SG profiles and diversity in PRs and ARs of seven *S*. *dulcamara* individuals. The individual plants were obtained by manual cross-pollination of two accessions with different chemotypes used in earlier studies (TW12 and ZD04; see Calf et al. 2018). The PRs and ARs were analyzed using liquid chromatography coupled with electrospray ionization quadruple Time-of-Flight mass spectrometry (LC-ESI-qToF-MS). By interpreting the resulting mass spectra, thereby zooming in on specific SGs masses, we found 31 SGs in total. We also assessed that ARs and PRs differ in their SGs profiles, which we discuss in relation to their functional role and ecological interactions.

## Materials and methods

### Plant material and experimental design

*Solanum dulcamara* plants were collected from different locations in The Netherlands and stored at Radboud University Genebank (https://www.ru.nl/bgard/) from where we obtained a seed batch (the collection is now maintained by the Centre for Genetic Resources, Wageningen, The Netherlands). *S. dulcamara* accessions “Texel Wet 12” (TW12) and “Zandvoort Dry 04” (ZD04) used in an earlier study, were brought to the greenhouse of the botanical garden of the University of Leipzig (Calf et al. 2018). Reciprocal crosses and selfings were manually made using accessions TW12 and ZD04. Table 1 summarizes the resulting four genotypes with the labels used to represent them. It also shows the numbers of individuals per genotype analyzed after seed germination.

**Table 1:** Greenhouse, manually produced genotypes of *Solanum dulcamara*

Pollen donor	Ovule donor	Resulting genotype	Number of individuals
TW12	ZD04	TW12xZD04 (crossed)	2
ZD04	TW12	ZD04xTW12 (crossed)	2
TW12	TW12	TW selfed	1
ZD04	ZD04	ZD selfed	2

Plants are descendants of wild individuals collected in Texel Wet (TW) and Zandvoort Dry (ZD) in The Netherlands

Germination of *S*. *dulcamara* seeds was induced based on the protocol described by Calf et al. (2018). Plastic boxes were filled with a layer of glass beads (1 mm Ø) onto which seeds were placed. Tap water was added up to the level of the beads and the seeds were cold-stratified at 4 °C in the dark for 2 weeks. Afterwards, the boxes were transferred to a climate chamber to induce germination under controlled conditions (photoperiod 16 h light/8 h dark, temperatures of 20 °C day/17 °C night and light intensity 500 μmol m^−2^ s^−1^). Emerged seedlings were transplanted to soil pots containing a 1:1 (v/v) mix of soil (Floradur B pot clay medium coarse, Floragard Vetriebs-GmbH, Germany) and sand (0/2 washed, Rösl Rohstoffe GmbH & Co. KG, Regensburg, Germany). The potted plants were grown under the previously described conditions for 4 months. Afterwards, they were removed from the pots and carefully washed with tap water to remove the soil surrounding the roots. Their stems were cut off at the base of the primary root and the dissected PRs were stored in Falcon tubes (Fisher Scientific GmbH) and stored at − 80 °C until used for compound extraction. The remaining stems were introduced in 50 mL Falcon tubes (Fisher Scientific GmbH) containing 40 mL of tap water immediately after dissection. The stems were kept in tap water in the climate chamber under the conditions described above to induce the growth of ARs. The water in the Falcon tube was exchanged weekly for 3 weeks. Plants were inspected regularly and the ARs that had grown to approx. 5 cm in length were dissected and frozen at − 80 °C. PRs and ARs were freeze-dried before compound extraction.

### Compound extraction

The freeze-dried samples were prepared following a procedure derived from De Vos et al. (2012) combined with that of Rogachev and Aharoni (2012). In brief, the extraction buffer was prepared by mixing 25% acetate buffer (2.3 mL acetic acid, 3.41 g ammonium acetate in 1 L millipore water, pH 4.8) with 75% methanol. For the PRs of individual plants, 20 mg of dried material was mixed with 1 mL extraction buffer in 2 mL Eppendorf tubes containing two metal beads (3 mm Ø). The tubes were then shaken for 10 min at 30 Hz in a TissueLyser (Qiagen N.V., Venlo, The Netherlands) and centrifuged for 15 min at 15,000 g. Then, 200 μL of the supernatant was transferred into LC–MS vials (1.5 mL ND9 bottle, Labsolute, Th. Geyer GmbH & Co. KG, Renningen, Germany) and dissolved in 800 μL extraction solution. The same protocol was followed for the ARs, although the volume of the extraction buffer was adjusted to the amount of root material obtained, which was notably lower. This ensured that the extraction efficiency among the two root types was similar and the resulting peak areas comparable among root types. For each plant, *ca*. 5 mg of freeze-dried roots was mixed with 500 μL buffer in 2 mL Eppendorf tubes and two metal beads. The tubes were shaken for 5 min at 30 Hz in a TissueLyser and centrifuged for 15 min at 15,000 g. Finally, 50 μL of the supernatant was transferred into LC–MS vials with glass inserts and dissolved in 100 μL extraction buffer.

### UPLC-qTOF-MS data acquisition

The plant extracts were analyzed using a UPLC-MS (Dionex UltiMate™ 3000, Thermo Fisher Scientific, Waltham, USA) equipped with a C18 analytical column (Acclaim TM RSLC 120; 2.1 × 150 mm, 2.2 µm particle size, 120 Å pore size). The column was kept at 40 °C. Mobile phase composition: solvent A: water/formic acid (0.05% v/v), solvent B: acetonitrile/formic acid (0.05% v/v). Flow rate: 400 μL min^–1^. The multi-step gradient for solvent B was: 0 − 1 min 5%, 1 − 4 min 28%, 4 − 10 min 36%, 10 − 12 min 95%, 12 − 14 min 95%, 14 − 16 min 5%, 16 − 18 min 5%. The chromatograph was equipped with an autosampler that kept the samples at a constant temperature of 4 °C and injected sample volumes of 10 μL.

The chromatograph was coupled with a maXis impact HD MS-qToF (Bruker Daltonics. Hamburg, Germany) operated in positive polarity. ESI source conditions were: end plate offset = 500 V, capillary = 4500 V, nebulizer = 2.5 bar, dry gas = 11 L min^−1^, dry temperature = 220 °C. Transfer line conditions were: funnels 1 and 2 = RF 300 Vpp, isCD energy = 0 eV, hexapole = 60 Vpp, quadrupole ion energy = 5 eV, low mass = 50 m/*z*, collision cell energy = 10 eV, collision RF 500 Vpp, transfer time = 60 µs, pre-pulse storage = 5 µs. The mass spectrometer operated in MS^1^ mode with a mass range of 50 − 1500 m/*z* and a spectral acquisition rate of 3 Hz. Sodium formate clusters (10 mM) were used for calibrating the *m*/*z* values. The clusters mix consisted of: 250 mL isopropanol, 1 mL formic acid, 5 mL 1 M sodium hydroxide and the final volume was adjusted to 500 mL.

All the chromatograms were uploaded to Zenodo (https://doi.org/10.5281/zenodo.6810952) (Chiocchio et al. 2022).

### UPLC-qTOF-MS data processing

The LC–MS files were processed with the program DataAnalysis 4.2 (Bruker Daltonics, Hamburg, Germany). Previous reports showed that in-source fragmentation of SGs produces protonated SAgls (Calf et al. 2018). Using this information, we obtained extracted ion chromatograms (EIC) for the *m*/*z* values 414.3372 and 416.3528 corresponding with the protonated adducts of the steroids dehydro-tomatidine/solasodine and tomatidine/soladulcidine, respectively. We overlaid the EIC of all the samples and manually compared the mass spectra of peaks with similar retention times. This comparison allowed us to assess the retention time stability necessary for comparing peak intensity for semi-quantitation among samples.

We manually interpreted mass spectra starting from the hypothesis that the mass spectra consist of *m/z* signals corresponding to the sequential fragmentation of glycoside moieties splitting off from the SGs. Our interpretation was supported by comparative analyses of the mass spectrum of a commercial glycoalkaloid, tomatine. This MS spectrum was obtained on the same platform under the same experimental conditions and reported in earlier studies (e.g. Mbaluto et al. 2021; Martínez-Medina et al. 2021). We found that in the same region of the chromatogram where the *m*/*z* values 414.3372 and 416.3528 occurred, other peaks following a comparable deglycosylation fragmentation pattern were present. By combining information on retention time and mass spectra, we were able to determine the total number of individual SGs for every chromatogram. For every SGs we selected a representative *m*/*z* value for semi-quantify compounds across samples. In order to maximize instrument sensitivity, we chose the highest *m*/*z* value in the mass spectrum of each compound (Table S1). In some cases, we chose a different representative *m*/*z* value when two closely eluting SGs were poorly resolved.

An EIC for every selected *m*/*z* value was produced and we obtained the integrals of the peaks in the EIC for quantitation. We visualized the presence and amounts/concentration by means of a bubble plot (Fig. 4) of the EIC integrals. To reduce overlapping bubbles, all integrals were divided by 2, keeping the proportionality in the final plot, thereby improving data visualization in the bubble plot. In order to profile the SG diversity across all the studied roots, we computed principal component analysis (PCA) using the integrals. The bubble plot was computed with Microsoft Excel (Version 2204, Build 16.0.15128.20210) and PCA (unit of variance (UV)-scaled) was computed using SIMCA 16 (Umetrics, Umea, Sweden).

## Results and discussion

### Detected SGs and classification

**Fig. 1:**
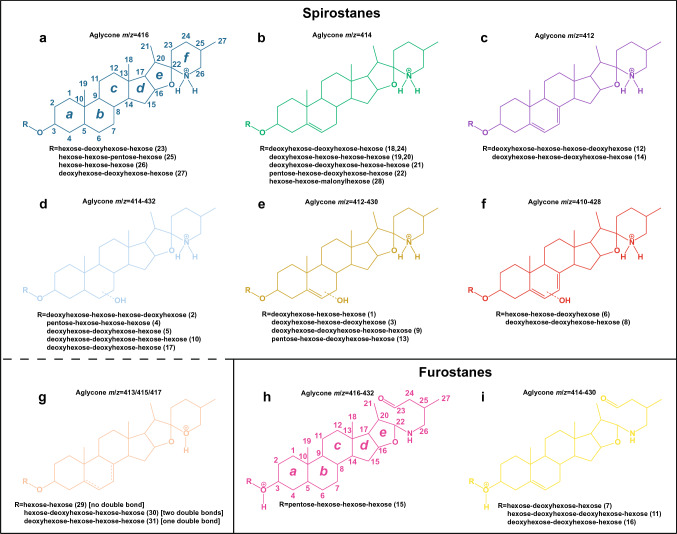
Proposed structures produced by interpreting the mass spectra of the 31 detected steroidal glycosides (SGs) in roots of *Solanum dulcamara*. Protonated adducts (X − H^⊕^) are shown as deducted from LC–qToF mass spectra. SGs are grouped into 9 classes (**a** − **i**) according to the steroidal aglycone (SAgl) type. For simplicity, the SAgl *m*/*z* values are shown only as integers. A detailed description of the retention times and *m*/*z* values for all the SGs is shown in supplementary Table S1. SAgls **a** to **g** are interpreted as spirostanes whereas SAgls **h** and **i** are interpreted as furostanes. IUPAC carbon atom numbering (1 − 27) and ring lettering (*a* − *f* or *a* − *e*) are shown for spirostanes and furostanes. The mass spectra of SGs **d** − **f** do not allow the exact position of the hydroxyl group to be determined (thus the bonds are represented as dotted lines). Color codes correspond with colors shown in Figs. 3, 4

By combining retention time and mass spectral properties, we found 31 different SGs in *S. dulcamara* PRs and ARs extracts (Table S1). Based on the interpretation of their mass spectra, we assigned the SGs to nine classes based on the *m*/*z* values of their SAgls (Fig. 1). In all the detected compounds, the *m*/*z* value attributed to the SAgl corresponds to a 27 carbon atom sterol, e.g. cholesterol. Modified triterpenes with a steroid core and 27 carbon atoms are commonly found in the genus *Solanum* (Perron and Albizati 1989). Of our proposed SAgls classes, seven fit structures with spirostane-like SAgls (Fig. 1a − g; 6-ringed) and two fit structures with furostane-like SAgls (Fig. 1h − i, 5-ringed) according to IUPAC nomenclature (Moss 1989).

### Spirostanes

In *S*. *dulcamara* and *Solanum lycopersicum* (tomato) many of the reported SAgls are spirostanes, that is, 6-ringed moieties with 27 carbons (Milner et al. 2011; Fig. 1). However, the exact SAgl structure of many SGs in *Solanum* species remains unresolved due to the lack of NMR or X-ray spectra. Nonetheless, we considered spirostanes as a likely structure for the SAgl we detected and used this as a starting point to assign different SG classes based on mass spectral interpretation.

Six of our proposed spirostane-like SAgls fit with the presence of a nitrogen atom in ring ***f ***(Fig. 1a − f) showing *m/z* values equal to 416.35, 414.33, and 412.32. These six classes can be divided into two sub-classes: SAgls without (Fig. 1; class **a, b, c)** and with (**d**, **e**, **f**) hydroxyl groups on the ***b*** ring. The presence of a hydroxyl group in SAgls **d**, **e** and **f** is supported by the presence of *m/z* signals equal to 432.35, 430.33, and 428.32, respectively. The difference between these three latter *m/z* values and the lighter ones corresponds to the loss of a water molecule (Δ*m* = 18.0105) and the concomitant formation of a double bond, which is common after losing a water molecule. The latter data corroborate an earlier study suggesting the presence of hydroxylated SAgls in root extracts from *S. dulcamara* (Willuhn and Kunanake 1970). SGs of classes **a** − **c** and **d** − **f** comprise a series of three SAgls with an increasing degree of unsaturation in the SAgl, which we illustrate by adding double bonds to ring ***b*** (Fig. 1, classes **c** and **f**).

The remaining class of spirostane-like SAgls, **g**, shows *m*/*z* values compatible with the presence of an oxygen atom in ring ***f*** (Fig. 1). In this SAgl class, we also found three SGs with varying degrees of unsaturation showing *m*/*z* values equal to 417.34, 415.32, and 413.30.

### Furostanes

**Fig. 2:**
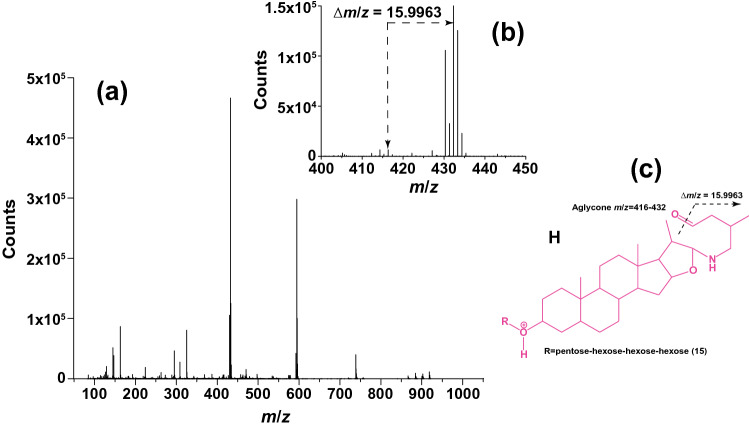
Mass spectrum of compound 15 (class H) showing an oxygen loss. This fragmentation supports the proposed SAgl structure consisting of a furostane bearing an aldehyde in the lateral chain

The *m*/*z* values of our two proposed furostane-like SAgls fit with the presence of a nitrogen atom and an aldehyde (C23) in the lateral chain at ring ***e***. The presence of an aldehyde is further supported by the presence of a fragment fitting with a mass loss equivalent to an oxygen atom. For example, the mass spectrum of compound 15, belonging to class **h**, showed *m/z* values 432.3479 and 416.3516 (Δ*m*/*z* = 15.9963 = 1 oxygen atom) (Fig. 2, Table S1).

We theorize that the oxygen loss is followed by the formation of a double bond between carbons 23 and 24 of the side chain. Mechanistically, this in-source fragmentation can be rationalized as a hydrodeoxygenation reaction, whereby an ion loses the equivalent of a molecule of water and gains the equivalent of a molecule of hydrogen for a net loss of one oxygen atom (− H_2_O + H_2_ =  − O; Eq. 1). Reactions comparable to hydrodeoxygenations of aldehydes have been long known, but hydrodeoxygenations in mass spectrometry are not commonly reported as fragmentation reactions (Dewar et al. 1981; Ahmed and Shevlin 1983). Stoichiometrically, the H_2_ molecule in the hydrodeoxygenation in Eq. 1 can result from the coordinated action of a proton and a hydride on a single substrate (here a furostane-like SAgls). Free protons commonly occur in positive mode LC–MS with an acidic mobile phase, like the conditions used in this study, and are thus readily available for a hydrodeoxygenation. However, the participation of a hydride is a less common mechanism in positive mode LC–MS fragmentation reactions. Whether the structural properties of the furostane-like SAgls and the interaction of these specific ions with the molecular matrix, either with the mobile phase or with other analytes, in electrospray ionization mass spectrometry favor this type of fragmentation, remains to be studied.

### Glycoside side chain

The analysis of the neutral losses derived from the mass spectra allowed us to classify the glycosidic units into hexoses, deoxyhexoses, and pentoses (Table S1). The exact type of sugar and the glycoside branching cannot be determined based on mass spectra, but we could readily identify di-, tri- and tetrasaccharides (Table S1). In addition, we also found *m*/*z* values in the mass spectra that we interpret as the presence of a malonylated hexose (Supplementary Table S1, compound 28).

In total, we assigned 31 structurally different putative steroidal glycosides which varied in the aglycon structure as well as in the glycoside moiety in root extracts of seven *S. dulcamara* individuals.

### AR and PR profile comparison

To investigate whether AR and PR differ in the SGs composition, we semi-quantified the compounds in each individual by integrating the extracted ion chromatogram (EIC) of a representative *m/z* value (in bold font in supplementary Table S1) for each compound. Subsequently, these values were subjected to principal component analysis (PCA) in order to facilitate the recognition of any data structure associated with the two types of roots studied.

**Fig. 3:**
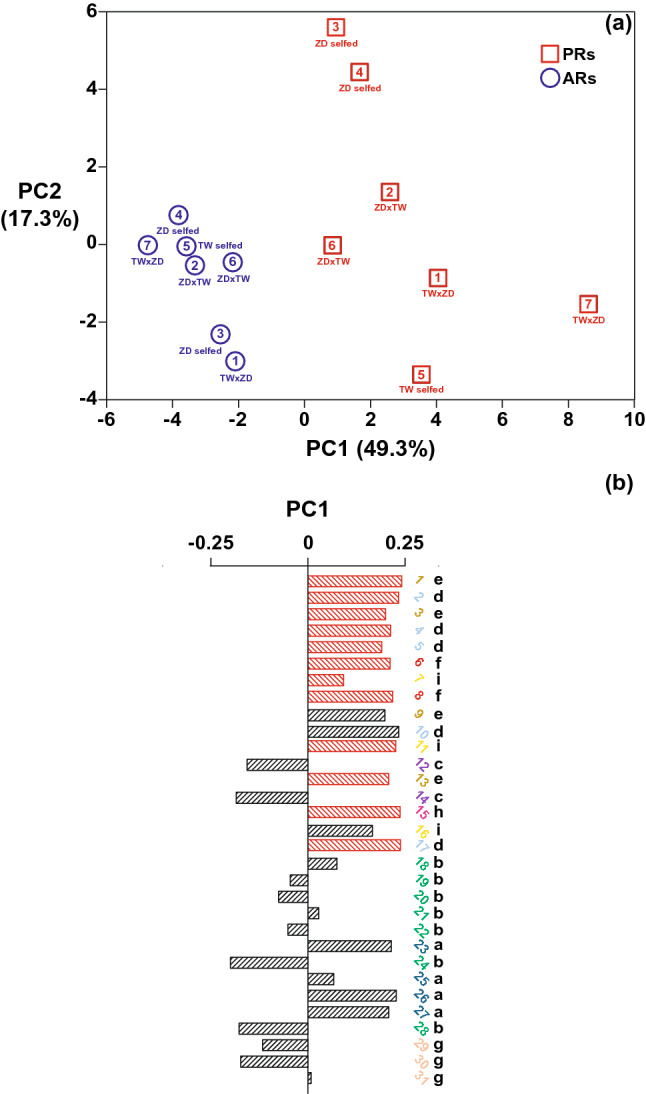
Principal component analysis (PCA) of the LC-qToF ion abundance (counts) as EIC integrals of the 31 glycosidic steroids (SGs) detected in roots of *Solanum dulcamara*. **a** Scores plot of primary (red squares -PRs-) and adventitious roots (blue circles -ARs-). Numbers 1 − 7 represent plant individuals, obtained from crossing or selfing of the two accessions TW12 and ZD04 (see Calf et al. 2018). **b** PC1 loadings for every EIC integral. Black bars represent SGs detected both in PRs and ARs, red bars represent SGs exclusively detected in PRs. Compound ID numbers (1 − 31) are shown in the color code for SAgl type (Figs. 1, 4)

The PCA scores plot shows that the SGA profiles of PRs and ARs separate along PC1 (Fig. 3a). Based on their PC1 loading scores, we grouped the *m*/*z* values into two types: *m*/*z* signals detected only in PRs and *m*/*z* signals common to both PRs and ARs (Fig. 3b). The PC1 loadings plot shows that for SGs present in both root types, the classes **c** (hypothetical ergosterol-like spirostanes) and **g** (hypothetical spirostanes with an oxygen bearing ring ***f***), display negative values, meaning they are more prominent in ARs. Qualitatively, the profiles of the PRs were richer, with more SGs detected than in the ARs. Three out of the four furostanes (classes **h** and **i**), and nine out of eleven SGs belonging to the SAgl classes **d** − **f** (hydroxylated spirostanes) were detected exclusively in the PRs. Overall, AR profiles of individual plants were more uniform, as they were less separated in the PCA plot (Fig. 3a).

**Fig. 4:**
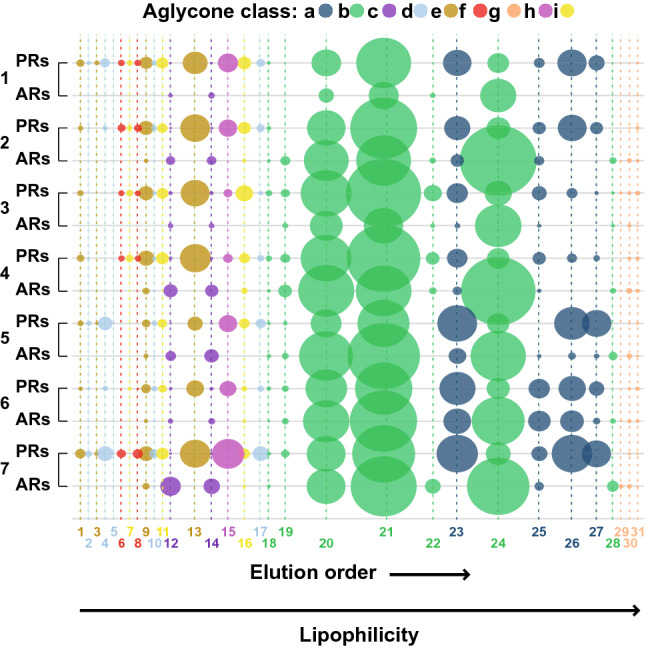
Bubble plot representing the LC–qToF ion abundance. Each bubble is proportional to the area of an *m*/*z* value representing each SG (EIC integrals). The compound number ID (1 − 31) corresponds with the elution order. Steroidal aglycone (SAgl) class and color codes correspond with those shown in Figs. 1, 3. Plant number (1 − 7) corresponds with Fig. 3a. *PRs* primary roots, *ARs* adventitious roots

To visualize differences in SGs profiles between roots on the same plant in a semi-quantitative manner, we generated pseudo-chromatograms in the form of a bubble plot (Fig. 4). The bubble plot shows the 31 SGs from left to right according to their elution order. This representation thus also visualizes the relative lipophilicity of the reported SGs. When paired per plant, this pseudo-chromatogram demonstrates that PRs and ARs have distinctive SG profiles (Fig. 4). The bubble plot also shows quantitative differences between the root types. For instance, ion abundances for compound 21 on average were larger in the PRs, whereas this pattern is inverted for compound 24. Both SGs are class **b** SAgls with different glycosides, suggesting that differential in situ biosynthesis or intra-organ transportation might underly these chemical differences.

Another example are SGs belonging to the SAgl classes **c** and **f**. Class **c** SGs are more abundant in ARs, while class **f** SGs have higher levels in the PRs. Our interpretation of the mass spectra of these two SGs classes suggests that they could be spirostanes bearing two double bonds. The difference between the two classes being a hydroxyl group present in class **f** SGs (Fig. 1).

As a pioneer study on *S. dulcamara* roots profiling, we focused on the comparison of two types of roots produced by the individuals. Whereas this ensures that AR and PR pairs come from a uniform genetic background, it also implies that they grew at different points in time. This might also have influenced the accumulation of the SGs, either due to differences in biosynthesis or transport activities. In *Solanum* species SGs biosynthesis primarily occurs in proliferating tissues, including the root apical meristems and leaf primordia. However the mechanism that control the SGs transport is not clear (Zhao et al. 2021).

## Concluding remarks and ecological interpretation

In conclusion**,** by analyzing *S. dulcamara* PRs and ARs, we identified 31 SGs, which we classified into 9 sub-classes based on their SAgls. By doing so, we discovered four putative SGs classes (**c**, **f**, **h**, **i**) new to *S. dulcamara*. Qualitative and quantitative comparisons showed that SGs profiles and concentrations differed between the two types of roots produced by this plant: PRs were richer in SGs than ARs, and the majority of the furostanes and hydroxylated spirostanes were present in PRs only. While we cannot determine the structural details of the SGs due to the lack of NMR or X-ray data, the structural features that we can derive from the mass spectra allow us to discuss the biochemistry and possible effects of the SGs chemodiversity in the ecology of *S. dulcamara*.

The most recent scientific literature describing *S. dulcamara* SGs mainly focuses on spirostane-like SGs such as dehydro-tomatidine/solasodine and their saturated aglycones tomatidine/soladulcidine. Profiling *S. dulcamara* extracts using LC–MS, we found evidence that the SGs profile of this plant is more complex including also additional double bonds and hydroxyl groups as well as furostane-like SGs. Both PRs and ARs produced SGs whose mass spectra fit with spirostanes with two double bonds, a feature we associate with the structure of ergosterol, a steroid described in fungi and protozoa. If further studies confirm that *S*. *dulcamara* roots contain ergosterol-like SGs, it would be important to determine whether the SGs are produced by a biosynthetic pathway similar to the one reported in tomato (Sonawane et al. 2016), or if the SGs are produced by symbionts associated with this plant.

We also detected sterols with double bonds. Recently, sterols bearing two double bonds in ring ***b*** were reported as intermediate of the cholesterol and phytosterol biosynthesis pathway in tomato (Sonawane et al. 2016; Li et al. 2022). Ergosterols are another class of C-27 sterols known to bear two double bonds in its ring ***b***. From a biosynthetic viewpoint this observation is interesting, since ergosterol is a sterol associated with cell membranes of fungi and protozoa (Weete et al. 2010; Dupont et al. 2012). Therefore, follow-up studies into potential symbionts of *S. dulcamara* may shed light on the biosynthesis of this SGs with ergosterol-like SAgl. Another explanation might be the expression of a plant dehydrogenases/reductases in below-ground tissues, analogous to the expression of dehydrogenases/reductases in above-ground tissues like in tomato (Sonawane et al. 2018).

In addition, we found mass spectra we interpret as furostane-like SGs. Studies on the biosynthesis of SGs in *Solanum* plants postulate that cholesterol is cyclized to form spirostanes (6-ringed steroids), whereafter the glycosylations take place (Itkin et al. 2013; Sonawane et al. 2018). The possible existence of furostane-like SGs (5-ringed steroids) open the possibility for the discovery of new, unknown steroid biosynthesis pathways in *Solanum dulcamara*.

An interesting observation is that the abundance of oxygen-containing steroids was higher in ARs. Considering that the ARs we analyzed were submerged in water, differences in molecular oxygen availability may have influenced the biosynthesis of SGs in this root type. It is known that free oxygen acts as a factor in different reactions across the biosynthesis of phytosteroids (Summons et al. 2006). However, the subsequent reactions leading to E and F-ring formation are not fully elucidated. As a consequence, whether the relative availability of molecular oxygen directly relates to the synthesis of oxygen-containing steroids is a topic that deserves to be explored in future studies.

All SGs we reported have a saponin structure (Challinor and De Voss 2013). If *S*. *dulcamara* roots would exude these saponins into the soil, as tomato plants do (Kirwa et al. 2018), their surfactant activities could influence the solubility of other root exudates affecting their diffusion across the soil. Likewise, the same surfactant effect could modify the assimilation of nutrients and semiochemicals, thereby influencing intra- or interspecific communication (Nakayasu et al. 2021). Furthermore, SGs can be bioactive themselves acting as regulators in allelopathy, herbivory or infection (Kirwa et al. 2018; Rial et al. 2018; Korenblum et al. 2020). In particular, unsaturated SGs are effective against aboveground pathogens (Sonawane et al. 2018) and slugs (Calf et al. 2018). In this study we found SGs with different degrees of unsaturation in roots. Roots are exposed to as many, if not more, enemies as shoots, whereby different root classes may be exposed to different levels of herbivory (Tsunoda and van Dam 2017). Therefore, the differences in AR and PR profiles may be meaningful, also in the light of plant performance, considering that *S. dulcamara* can reproduce asexually via the production of stolons and ARs. It is, therefore, relevant to understand the chemistry of ARs to better assess its impact on root functional and chemical ecology.

The results presented here open new venues to evaluate the bioactivity of these molecules in belowground ecosystems. Given the many biological activities reported for these compounds, describing the chemodiversity of SGs may help to better understand the chemical ecology of SGs, as well as support potential application for human health. Further studies with larger cohorts and multiple samplings during the ontogeny of both PRs and ARs are needed to elucidate their function in natural ecosystems.

### *Author contribution statement*

FV and NMvD designed the study; NPA conducted the experiment; IC took the lead in processing the data with the contribution of FV and RAA; RAA was responsible for data archiving on Zenodo; FV, IC and NMvD wrote the paper. All authors read and approved the manuscript.

## Supplementary Information

Below is the link to the electronic supplementary material.

Supplementary file1 (DOCX 140 KB)

## Data Availability

The dataset generated during the current study is available in the Zenodo repository [https://zenodo.org/record/6810952#.YzXLpkxBz3k].
