# Supplementary material for: Steroidal glycoside profile differences among primary roots system and adventitious roots in Solanum dulcamara
Source: Planta. 2023 Jan 16;257(2):37. doi: 10.1007/s00425-023-04072-9 (PMC9842586; doi:10.1007/s00425-023-04072-9)
Supplement: Supplementary file 1 — Supplementary file1 (DOCX 140 KB) [file 425_2023_4072_MOESM1_ESM.docx]

**Table S1.** Summary of compounds detected by LC-qToF-MS in roots of *S*. *dulcamara*. Each compound was given an identification number according to its retention time and was classified according to the hypothetical aglycone (details shown on Figure 1). For each compound the *m*/*z* value used for quantitation (bubble plot, PCA) is highlighted in bold font. RT=retention time [s], PRs=Primary roots, ARs=Adventitious roots, Agly=aglycone, Hex=hexose, DeOxHex=deoxy-hexose, Pent=pentose.

| Hypothetical compound ID number, class, RT [s] and root type | Measured monoisotopic mass | Chemical formula | Intensity [counts] | Putative chemical fragmentation (M=molecular ion) | Putative annotation of chemical fragmentation (M=molecular ion) | Detected ion description |  |
| --- | --- | --- | --- | --- | --- | --- | --- |
| 1, E, 279.3, PRs | 900.5003 | C_45_H_74_NO_17_+ | 2028 | [M+H]^+^ | M | Putative molecular ion | |
|  | 754.4416 | C_39_H_64_NO_13_+ | 864 | [M-C_6_H_10_O_4_]^+^ | M-DeOxHex | Diglycosidic steroid | |
|  | 738.4457 | C_39_H_64_NO_12_+ | 712 | [M-C_6_H_10_O_5_]^+^ | M-Hex | Diglycosidic steroid | |
|  | 592.3860 | C_33_H_54_NO_8_+ | 29918 | [M-C_6_H_10_O_5_-C_6_H_10_O_4_]^+^ | M-Hex-DeOxHex | Monoglycosidic steroid | |
|  | **430.3323** | C_27_H_44_NO_3_+ | 80756 | [M-C_6_H_10_O_5_-C_6_H_10_O_4_-C_6_H_10_O_5_]^+^ | M-Hex-DeOxHex-Hex | Aglycone | |
|  | 412.3208 | C_27_H_42_NO_2_+ | 4868 | [M-C_6_H_10_O_5_-C_6_H_10_O_4_-C_6_H_10_O_5_-H_2_O]^+^ | M-Hex-DeOxHex-Hex-H_2_O | Aglycone fragment | |
|  | 325.1115 | C_12_H_21_O_10_+ | 3686 | [M-C_27_H_44_NO_3_-C_6_H_10_O_4_+H]^+^ | M-Agly-DeOxHex | Disaccharide (Hex-Hex) | |
|  | 289.0930 | C_12_H_17_O_8_+ | 664 | [M-C_27_H_44_NO_3_-C_6_H_10_O_4_-2H_2_O+H]^+^ | M-Agly-DeOxHex-2H_2_O |  | |
|  | 253.0700 | C_12_H_13_O_6_+ | 360 | [M-C_27_H_44_NO_3_-C_6_H_10_O_4_-4H_2_O+H]^+^ | M-Agly-DeOxHex-4H_2_O |  | |
|  | 309.1186 | C_12_H_21_O_9_+ | 11314 | [M-C_27_H_44_NO_3_-C_6_H_10_O_5_+H]^+^ | M-Agly-Hex | Disaccharide (DeOxHex-Hex) | |
|  | 291.1078 | C_12_H_19_O_8_+ | 982 | [M-C_27_H_44_NO_3_-C_6_H_10_O_5_-H_2_O+H]^+^ | M-Agly-Hex-H_2_O |  | |
|  | 273.0969 | C_12_H_17_O_7_+ | 3318 | [M-C_27_H_44_NO_3_-C_6_H_10_O_5_-2H_2_O+H]^+^ | M-Agly-Hex-2H_2_O |  | |
|  | 255.0876 | C_12_H_15_O_6_+ | 1182 | [M-C_27_H_44_NO_3_-C_6_H_10_O_5_-3H_2_O+H]^+^ | M-Agly-Hex-3H_2_O |  | |
|  | 163.0612 | C_6_H_11_O_5_+ | 7222 | [M-C_27_H_44_NO_3_-C_6_H_10_O_5_-C_6_H_10_O_4_+H]^+^ | M-Agly-Hex-DeOxHex | Hexose fragment | |
|  | 147.0659 | C_6_H_11_O_4_+ | 16932 | [M-C_27_H_44_NO_3_-C_6_H_10_O_5_-C_6_H_10_O_5_+H]^+^ | M-Agly-Hex-Hex | Deoxyhexose fragment | |
|  |  |  |  |  |  |  | |
| 2, D, 282, PRs | 1048.5705 | C_51_H_86_NO_21_+ | 104 | [M+H]^+^ | M | Putative molecular ion | |
|  | 902.5135 | C_45_H_76_NO_17_+ | 1872 | [M-C_6_H_10_O_4_]^+^ | M-DeOxHex | Triglycosidic steroid | |
|  | 756.4542 | C_39_H_66_NO_13_+ | 754 | [M-C_6_H_10_O_4_-C_6_H_10_O_4_]^+^ | M-DeOxHex-DeOxHex | Diglycosidic steroid | |
|  | 740.4579 | C_39_H_66_NO_12_+ | 824 | [M-C_6_H_10_O_4_-C_6_H_10_O_5_]^+^ | M-DeOxHex-Hex | Diglycosidic steroid | |
|  | 594.4011 | C_33_H_56_NO_8_+ | 22900 | [M-C_6_H_10_O_4_-C_6_H_10_O_4_-C_6_H_10_O_5_]^+^ | M-DeOxHex-DeOxHex-Hex | Monoglycosidic steroid | |
|  | **432.3475** | C_27_H_46_NO_3_+ | 71690 | [M-C_6_H_10_O_4_-C_6_H_10_O_4_-C_6_H_10_O_5_-C_6_H_10_O_5_]^+^ | M-DeOxHex-DeOxHex-Hex-Hex | Aglycone | |
|  | 414.3373 | C_27_H_44_NO_2_+ | 4354 | [M-C_6_H_10_O_4_-C_6_H_10_O_4_-C_6_H_10_O_5_-C_6_H_10_O_5_-H_2_O]+ | M-DeOxHex-DeOxHex-Hex-Hex-H_2_O | Aglycone fragment | |
|  | 309.1181 | C_12_H_21_O_9_+ | 13430 | [M-C_27_H_46_NO_3_-C_6_H_10_O_5_-C_6_H_10_O_4_+H]^+^ | M-Agly-Hex-DeOxHex | Disaccharide (DeOxHex-Hex) | |
|  | 291.1073 | C_12_H_19_O_8_+ | 810 | [M-C_27_H_46_NO_3_-C_6_H_10_O_5_-C_6_H_10_O_4_-H_2_O+H]^+^ | M-Agly-Hex-DeOxHex-H_2_O |  | |
|  | 273.0996 | C_12_H_17_O_7_+ | 3514 | [M-C_27_H_46_NO_3_-C_6_H_10_O_5_-C_6_H_10_O_4_-2H_2_O+H]^+^ | M-Agly-Hex-DeOxHex-2H_2_O |  | |
|  | 255.0860 | C_12_H_15_O_6_+ | 2104 | [M-C_27_H_46_NO_3_-C_6_H_10_O_5_-C_6_H_10_O_4_-3H_2_O+H]^+^ | M-Agly-Hex-DeOxHex-3H_2_O |  | |
|  | 237.0768 | C_12_H_13_O_5_+ | 402 | [M-C_27_H_46_NO_3_-C_6_H_10_O_5_-C_6_H_10_O_4_-4H_2_O+H]^+^ | M-Agly-Hex-DeOxHex-4H_2_O |  | |
|  | 147.0655 | C_6_H_11_O_4_+ | 19716 | [M-C_27_H_46_NO_3_-C_6_H_10_O_5_-C_6_H_10_O_5_-C_6_H_10_O_4_+H]^+^ | M-Agly-Hex-Hex-DeOxHex | Deoxyhexose fragment | |
|  |  |  |  |  |  |  | |
| 3, E, 285.5, PRs | 884.5029 | C_45_H_74_NO_16_+ | 17616 | [M+H]^+^ | M | Putative molecular ion | |
|  | 738.4423 | C_39_H_64_NO_12_+ | 1708 | [M-C_6_H_10_O_4_]^+^ | M-DeOxHex | Diglycosidic steroid | |
|  | **592.3864** | C_33_H_54_NO_8_+ | 35660 | [M-C_6_H_10_O_4_-C_6_H_10_O_4_]^+^ | M-DeOxHex-DeOxHex | Monoglycosidic steroid | |
|  | 430.3320 | C_27_H_44_NO_3_+ | 24522 | [M-C_6_H_10_O_4_-C_6_H_10_O_5_-C_6_H_10_O_4_]^+^ | M-DeOxHex-Hex-DeOxHex | Aglycone | |
|  | 412.3221 | C_27_H_42_NO_2_+ | 1726 | [M-C_6_H_10_O_4_ -C_6_H_10_O_5_-C_6_H_10_O_4_-H_2_O]^+^ | M-DeOxHex-Hex-DeOxHex-H_2_O | Aglycone fragment | |
|  | 309.1183 | C_12_H_21_O_9_+ | 2664 | [M-C_27_H_44_NO_3_-C_6_H_10_O_4_+H]^+^ | M-Agly-DeOxHex | Disaccharide (DeOxHex-Hex) | |
|  | 273.0963 | C_12_H_17_O_7_+ | 994 | [M-C_27_H_44_NO_3_-C_6_H_10_O_4_-2H_2_O+H]^+^ | M-Agly-DeOxHex-2H_2_O |  | |
|  | 255.0865 | C_12_H_15_O_6_+ | 990 | [M-C_27_H_44_NO_3_-C_6_H_10_O_4_-3H_2_O+H]^+^ | M-Agly-DeOxHex-3H_2_O |  | |
|  | 237.0765 | C_12_H_13_O_5_+ | 318 | [M-C_27_H_44_NO_3_-C_6_H_10_O_4_-4H_2_O+H]^+^ | M-Agly-DeOxHex-4H_2_O |  | |
|  | 163.0607 | C_6_H_11_O_5_+ | 22606 | [M-C_27_H_44_NO_3_ -C_6_H_10_O_4_-C_6_H_10_O_4_-H_2_O+H]^+^ | M-Agly-DeOxHex-DeOxHex-H_2_O | Hexose fragment | |
|  | 147.0656 | C_6_H_11_O_4_+ | 7908 | [M-C_27_H_44_NO_3_-C_6_H_10_O_4_-C_6_H_10_O_5_-H_2_O+H]^+^ | M-Agly-DeOxHex-Hex-H_2_O | Deoxyhexose fragment | |
|  |  |  |  |  |  |  | |
| 4, D, 286, PRs | 1050.5513 | C_50_H_84_NO_22_+ | 94 | [M+H]^+^ | M | Putative molecular ion | |
|  | 918.5073 | C_45_H_76_NO_18_+ | 10366 | [M-C_5_H_8_O_4_]^+^ | M-Pent | Triglycosidic steroid | |
|  | 756.4536 | C_39_H_66_NO_13_+ | 1130 | [M-C_5_H_8_O_4_-C_6_H_10_O_5_]^+^ | M-Pent-Hex | Diglycosidic steroid | |
|  | 738.4470 | C_39_H_64_NO_12_+ | 1160 | [M-C_5_H_8_O_4_-C_6_H_10_O_5_-H_2_O]^+^ | M-Pent-Hex-H_2_O | Diglycosidic steroid | |
|  | 594.4021 | C_33_H_56_NO_8_+ | 192774 | [M-C_5_H_8_O_4_ -C_6_H_10_O_5_-C_6_H_10_O_5_]^+^ | M-Pent-Hex-Hex | Monoglycosidic steroid | |
|  | 486.1555 | C_18_H_30_O_15_+ | 66 | [M-C_27_H_46_NO_3_-C_5_H_8_O_4_]^+^ | M-Agly-Pent | Trisaccharide (Hex-Hex-Hex) | |
|  | 457.1553 | C_17_H_29_O_14_+ | 594 | [M-C_27_H_46_NO_3_-C_6_H_10_O_5_+H]^+^ | M-Agly-Hex+H | Trisaccharide (Pent-Hex-Hex) | |
|  | **432.3482** | C_27_H_46_NO_3_+ | 322840 | [M-C_5_H_8_O_4_-C_6_H_10_O_5_-C_6_H_10_O_5_-C_6_H_10_O_5_ ]^+^ | M-Pent-Hex-Hex-Hex | Aglycone | |
|  | 414.3366 | C_27_H_44_NO_2_+ | 9362 | [M-C_5_H_8_O_4_-C_6_H_10_O_5_-C_6_H_10_O_5_-C_6_H_10_O_5_ -H_2_O]^+^ | M-Pent-Hex-Hex-Hex-H_2_O | Aglycone fragment | |
|  | 325.1132 | C_12_H_21_O_10_+ | 83698 | [M-C_27_H_46_NO_3_-C_5_H_8_O_4_-C_6_H_10_O_5_]^+^ | M-Agly-Pent-Hex | Disaccharide (Hex-Hex) | |
|  | 307.1026 | C_12_H_19_O_9_+ | 828 | [M-C_27_H_46_NO_3_-C_5_H_8_O_4_-C_6_H_10_O_5_-H_2_O+H]^+^ | M-Agly-Pent-Hex-H_2_O |  | |
|  | 289.0913 | C_12_H_17_O_8_+ | 6934 | [M-C_27_H_46_NO_3_-C_5_H_8_O_4_-C_6_H_10_O_5_-2H_2_O+H]^+^ | M-Agly-Pent-Hex-2H_2_O |  | |
|  | 271.0817 | C_12_H_15_O_7_+ | 1640 | [M-C_27_H_46_NO_3_-C_5_H_8_O_4_-C_6_H_10_O_5_-3H_2_O+H]^+^ | M-Agly-Pent-Hex-3H_2_O |  | |
|  | 253.0699 | C_12_H_13_O_6_+ | 688 | [M-C_27_H_46_NO_3_-C_5_H_8_O_4_-C_6_H_10_O_5_-4H_2_O+H]^+^ | M-Agly-Pent-Hex-4H_2_O |  | |
|  | 163.0608 | C_6_H_11_O_5_+ | 114604 | [M-C_27_H_46_NO_3_-C_5_H_8_O_4_-C_6_H_10_O_5_-C_6_H_10_O_5_+H]^+^ | M-Agly-Pent-Hex-Hex | Hexose fragment | |
|  | 145.0500 | C_6_H_9_O_4_+ | 61498 | [M-C_27_H_46_NO_3_-C_5_H_8_O_4_ -C_6_H_10_O_5_-C_6_H_10_O_5_-H_2_O+H]^+^ | M-Agly-Pent-Hex-Hex-H_2_O |  | |
|  | 133.0495 | C_5_H_9_O_4_+ | 836 | [M-C_27_H_46_NO_3_-C_6_H_10_O_5_ -C_6_H_10_O_5_-C_6_H_10_O_5_+H]^+^ | M-Agly-Hex-Hex-Hex | Pentose fragment | |
|  |  |  |  |  |  |  | |
| 5, D, 289.6, PRs | **886.5205** | C_45_H_76_NO_16_+ | 6268 | [M+H]^+^ | M | Putative molecular ion | |
|  | 740.4609 | C_39_H_66_NO_12_+ | 3142 | [M-C_6_H_10_O_4_]^+^ | M-DeOxHex | Diglycosidic steroid | |
|  | 594.4022 | C_33_H_56_NO_8_+ | 34976 | [M-C_6_H_10_O_4_-C_6_H_10_O_4_]^+^ | M-DeOxHex-DeOxHex | Monoglycosidic steroid | |
|  | 432.3478 | C_27_H_46_NO_3_+ | 45484 | [M-C_6_H_10_O_4_-C_6_H_10_O_4_-C_6_H_10_O_5_]^+^ | M-DeOxHex-DeOxHex-Hex | Aglycone | |
|  | 414.3371 | C_27_H_44_NO_2_+ | 11010 | [M-C_6_H_10_O_4_-C_6_H_10_O_4_-C_6_H_10_O_5_-H_2_O]^+^ | M-DeOxHex-DeOxHex-Hex-H_2_O | Aglycone fragment | |
|  | 291.1075 | C_12_H_19_O_8_+ | 234 | [M-C_27_H_46_NO_3_-C_6_H_10_O_4_-H_2_O+H]^+^ | M-Agly-DeOxHex-H_2_O |  | |
|  | 273.0971 | C_12_H_17_O_7_+ | 1034 | [M-C_27_H_46_NO_3_-C_6_H_10_O_4_-2H_2_O+H]^+^ | M-Agly-DeOxHex-2H_2_O |  | |
|  | 255.0865 | C_12_H_15_O_6_+ | 560 | [M-C_27_H_46_NO_3_-C_6_H_10_O_4_-3H_2_O+H]^+^ | M-Agly-DeOxHex-3H_2_O |  | |
|  | 163.0604 | C_6_H_11_O_5_+ | 22366 | [M-C_27_H_46_NO_3_-C_6_H_10_O_4_-C_6_H_10_O_4_+H]^+^ | M-Agly-DeOxHex-DeOxHex | Hexose fragment | |
|  | 147.0657 | C_6_H_11_O_4_+ | 4176 | [M-C_27_H_46_NO_3_-C_6_H_10_O_4_-C_6_H_10_O_5_+H]^+^ | M-Agly-DeOxHex-Hex | Deoxyhexose fragment | |
|  |  |  |  |  |  |  | |
| 6, F, 292.3, PRs | 898.4836 | C_45_H_72_NO_17_+ | 2512 | [M+H]^+^ | M | Putative molecular ion | |
|  | 752.4263 | C_39_H_62_NO_13_+ | 1652 | [M-C_6_H_10_O_4_]^+^ | M-DeOxHex | Diglycosidic steroid | |
|  | 736.4282 | C_39_H_62_NO_12_+ | 2208 | [M-C_6_H_10_O_5_]^+^ | M-Hex | Diglycosidic steroid | |
|  | 590.3700 | C_33_H_52_NO_8_+ | 29564 | [M-C_6_H_10_O_4_-C_6_H_10_O_5_]^+^ | M-DeOxHex-Hex | Monoglycosidic steroid | |
|  | **428.3166** | C_27_H_42_NO_3_+ | 107086 | [M-C_6_H_10_O_4_-C_6_H_10_O_5_-C_6_H_10_O_4_]^+^ | M-DeOxHex-Hex-DeOxHex | Aglycone | |
|  | 410.3045 | C_27_H_40_NO_2_+ | 902 | [M-C_6_H_10_O_4_-C_6_H_10_O_5_-C_6_H_10_O_4_-H_2_O]^+^ | M-DeOxHex-Hex-DeOxHex-H_2_O | Aglycone fragment | |
|  | 309.1183 | C_12_H_21_O_9_+ | 15764 | [M-C_27_H_42_NO_3_-C_6_H_10_O_5_+H]^+^ | M-Agly-Hex | Disaccharide (DeOxHex-Hex) | |
|  | 291.1100 | C_12_H_19_O_8_+ | 1324 | [M-C_27_H_42_NO_3_-C_6_H_10_O_5_-H_2_O+H]^+^ | M-Agly-Hex-H_2_O |  | |
|  | 273.0975 | C_12_H_17_O_7_+ | 5178 | [M-C_27_H_42_NO_3_-C_6_H_10_O_5_-2H_2_O+H]^+^ | M-Agly-Hex-2H_2_O |  | |
|  | 255.0871 | C_12_H_15_O_6_+ | 2564 | [M-C_27_H_42_NO_3_-C_6_H_10_O_5_-3H_2_O+H]^+^ | M-Agly-Hex-3H_2_O |  | |
|  | 237.0761 | C_12_H_13_O_5_+ | 1066 | [M-C_27_H_42_NO_3_-C_6_H_10_O_5_-4H_2_O+H]^+^ | M-Agly-Hex-4H_2_O |  | |
|  | 147.0657 | C_6_H_11_O_4_+ | 25718 | [M-C_27_H_42_NO_3_-C_6_H_10_O_4_-C_6_H_10_O_5_+H]^+^ | M-Agly-DeOxHex-Hex | Deoxyhexose fragment | |
|  | 129.0550 | C_6_H_9_O_3_+ | 15120 | [M-C_27_H_42_NO_3_-C_6_H_10_O_4_-C_6_H_10_O_5_-H_2_O+H]^+^ | M-Agly-DeOxHex-Hex-H_2_O |  | |
|  |  |  |  |  |  |  | |
| 7, I, 297, PRs | 900.4970 | C_45_H_74_NO_17_+ | 3160 | [M+H]^+^ | M | Putative molecular ion | |
|  | 738.4432 | C_39_H_64_NO_12_+ | 1186 | [M-C_6_H_10_O_5_]^+^ | M-Hex | Diglycosidic steroid | |
|  | 592.3852 | C_33_H_54_NO_8_+ | 24682 | [M-C_6_H_10_O_5_-C_6_H_10_O_4_]^+^ | M-Hex-DeOxHex | Monoglycosidic steroid | |
|  | **430.3321** | C_27_H_44_NO_3_+ | 94594 | [M-C_6_H_10_O_5_-C_6_H_10_O_4_-C_6_H_10_O_5_]^+^ | M-Hex-DeOxHex-Hex | Aglycone | |
|  | 414.3376 | C_27_H_44_NO_2_+ | 2982 | [M-C_6_H_10_O_5_-C_6_H_10_O_4_-C_6_H_10_O_5_-O]^+^ | M-Hex-DeOxHex-Hex-Ox | Aglycone fragment | |
|  | 325.1125 | C_12_H_21_O_10_+ | 6584 | [M-C_27_H_44_NO_3_-C_6_H_10_O_4_+H]^+^ | M-Agly-DeOxHex | Disaccharide (Hex-Hex) | |
|  | 309.1180 | C_12_H_21_O_9_+ | 11368 | [M-C_27_H_44_NO_3_-C_6_H_10_O_5_+H]^+^ | M-Agly-Hex | Disaccharide (DeOxHex-Hex) | |
|  | 163.0606 | C_6_H_11_O_5_+ | 10898 | [M-C_27_H_44_NO_3_-C_6_H_10_O_4_-C_6_H_10_O_5_+H]^+^ | M-Agly-DeOxHex-Hex | Hexose fragment | |
|  | 147.0655 | C_6_H_11_O_4_+ | 22944 | [M-C_27_H_44_NO_3_-C_6_H_10_O_4_-C_6_H_10_O_4_+H]^+^ | M-Agly-DeOxHex-DeOxHex | Deoxyhexose fragment | |
|  | 129.0550 | C_6_H_9_O_3_+ | 13126 | [M-C_27_H_44_NO_3_-C_6_H_10_O_4_-C_6_H_10_O_4_-H_2_O+H]^+^ | M-Agly-DeOxHex-DeOxHex-H_2_O |  | |
|  |  |  |  |  |  |  | |
| 8, F, 299.9, PRs | 882.4867 | C_45_H_72_NO_16_+ | 55144 | [M+H]^+^ | M | Putative molecular ion | |
|  | 736.4295 | C_39_H_62_NO_12_+ | 33590 | [M-C_6_H_10_O_4_]^+^ | M-DeOxHex | Diglycosidic steroid | |
|  | **590.3706** | C_33_H_52_NO_8_+ | 111770 | [M-C_6_H_10_O_4_-C_6_H_10_O_4_]^+^ | M-DeOxHex-DeOxHex | Monoglycosidic steroid | |
|  | 428.3164 | C_27_H_42_NO_3_+ | 83862 | [M-C_6_H_10_O_4_-C_6_H_10_O_4_-C_6_H_10_O_5_]^+^ | M-DeOxHex-DeOxHex-Hex | Aglycone | |
|  | 410.3059 | C_27_H_40_NO_2_+ | 1002 | [M-C_6_H_10_O_4_-C_6_H_10_O_4_-C_6_H_10_O_5_-H_2_O]^+^ | M-DeOxHex-DeOxHex-Hex-H_2_O | Aglycone fragment | |
|  | 309.1181 | C_12_H_21_O_9_+ | 16978 | [M-C_27_H_42_NO_3_-C_6_H_10_O_4_+H]^+^ | M-Agly-DeOxHex | Disaccharide (DeOxHex-Hex) | |
|  | 293.1233 | C_12_H_21_O_8_+ | 36146 | [M-C_27_H_42_NO_3_-C_6_H_10_O_5_+H]^+^ | M-Agly-Hex | Disaccharide (DeOxHex-DeOxHex) | |
|  | 291.1075 | C_12_H_19_O_8_+ | 896 | [M-C_27_H_42_NO_3_-C_6_H_10_O_4_-H_2_O+H]^+^ | M-Agly-DeOxHex-H_2_O |  | |
|  | 275.1124 | C_12_H_19_O_7_+ | 14066 | [M-C_27_H_42_NO_3_-C_6_H_10_O_5_-H_2_O+H]^+^ | M-Agly-Hex-H_2_O |  | |
|  | 273.0965 | C_12_H_17_O_7_+ | 7142 | [M-C_27_H_42_NO_3_-C_6_H_10_O_4_-2H_2_O+H]^+^ | M-Agly-DeOxHex-2H_2_O |  | |
|  | 257.1029 | C_12_H_17_O_6_+ | 9116 | [M-C_27_H_42_NO_3_-C_6_H_10_O_5_-2H_2_O+H]^+^ | M-Agly-Hex-2H_2_O |  | |
|  | 255.0859 | C_12_H_15_O_6_+ | 3026 | [M-C_27_H_42_NO_3_-C_6_H_10_O_4_-3H_2_O+H]^+^ | M-Agly-DeOxHex-3H_2_O |  | |
|  | 239.0919 | C_12_H_15_O_5_+ | 11700 | [M-C_27_H_42_NO_3_-C_6_H_10_O_5_-3H_2_O+H]^+^ | M-Agly-Hex-3H_2_O |  | |
|  | 237.0753 | C_12_H_13_O_5_+ | 1726 | [M-C_27_H_42_NO_3_-C_6_H_10_O_4_-4H_2_O+H]^+^ | M-Agly-DeOxHex-4H_2_O |  | |
|  | 221.0818 | C_12_H_13_O_4_+ | 554 | [M-C_27_H_42_NO_3_-C_6_H_10_O_5_-4H_2_O+H]^+^ | M-Agly-Hex-4H_2_O |  | |
|  | 163.0606 | C_6_H_11_O_5_+ | 16378 | [M-C_27_H_42_NO_3_-C_6_H_10_O_4_-C_6_H_10_O_5_+H]^+^ | M-Agly-DeOxHex-Hex | Hexose fragment | |
|  | 147.0658 | C_6_H_11_O_4_+ | 38620 | [M-C_27_H_42_NO_3_-C_6_H_10_O_4_-C_6_H_10_O_4_+H]^+^ | M-Agly-DeOxHex-DeOxHex | Deoxyhexose fragment | |
|  | 129.0550 | C_6_H_9_O_3_+ | 23060 | [M-C_27_H_42_NO_3_-C_6_H_10_O_4_-C_6_H_10_O_4_-H_2_O+H]^+^ | M-Agly-DeOxHex-DeOxHex-H_2_O |  | |
|  |  |  |  |  |  |  | |
| 9, E, 301.9, PRs/ARs | 1046.5514 | C_51_H_84_NO_21_+ | 396 | [M+H]^+^ | M | Putative molecular ion | |
|  | 900.4964 | C_45_H_74_NO_17_+ | 23968 | [M-C_6_H_10_O_4_]^+^ | M-DeOxHex | Triglycosidic steroid | |
|  | 754.4401 | C_39_H_64_NO_13_+ | 3148 | [M-C_6_H_10_O_4_-C_6_H_10_O_4_]^+^ | M-DeOxHex-Hex | Diglycosidic steroid | |
|  | 738.4418 | C_39_H_64_NO_12_+ | 22104 | [M-C_6_H_10_O_4_-C_6_H_10_O_5_]^+^ | M-DeOxHex-DeOxHex | Diglycosidic steroid | |
|  | 592.3862 | C_33_H_54_NO_8_+ | 188376 | [M-C_6_H_10_O_4_-C_6_H_10_O_4_-C_6_H_10_O_5_]^+^ | M-DeOxHex-DeOxHex-Hex | Monoglycosidic steroid | |
|  | **430.3323** | C_27_H_44_NO_3_+ | 473192 | [M-C_6_H_10_O_4_-C_6_H_10_O_4_-C_6_H_10_O_5_-C_6_H_10_O_5_]^+^ | M-DeOxHex-DeOxHex-Hex-Hex | Aglycone | |
|  | 412.3224 | C_27_H_42_NO_2_+ | 2004 | [M-C_6_H_10_O_4_-C_6_H_10_O_4_-C_6_H_10_O_5_-C_6_H_10_O_5_-H_2_O]^+^ | M-DeOxHex-DeOxHex-Hex-Hex-H_2_O | Aglycone fragment | |
|  | 325.1121 | C_12_H_21_O_10_+ | 12706 | [M-C_27_H_44_NO_3_-C_6_H_10_O_4_-C_6_H_10_O_4_+H]^+^ | M-Agly-DeOxHex-DeOxHex | Disaccharide (Hex-Hex) | |
|  | 309.1182 | C_12_H_21_O_9_+ | 50246 | [M-C_27_H_44_NO_3_-C_6_H_10_O_4_-C_6_H_10_O_5_+H]^+^ | M-Agly-DeOxHex-Hex | Disaccharide (DeOxHex-Hex) | |
|  | 307.1042 | C_12_H_19_O_9_+ | 236 | [M-C_27_H_44_NO_3_-C_6_H_10_O_4_-C_6_H_10_O_4_-H_2_O+H]^+^ | M-Agly-DeOxHex-DeOxHex-H_2_O |  | |
|  | 293.1234 | C_12_H_21_O_8_+ | 27028 | [M-C_27_H_44_NO_3_-C_6_H_10_O_5_-C_6_H_10_O_5_+H]^+^ | M-Agly-Hex-Hex | Disaccharide (DeOxHex-DeOxHex) | |
|  | 291.1072 | C_12_H_19_O_8_+ | 2986 | [M-C_27_H_44_NO_3_-C_6_H_10_O_4_-C_6_H_10_O_5_-H_2_O+H]^+^ | M-Agly-DeOxHex-Hex-H_2_O |  | |
|  | 289.0916 | C_12_H_17_O_8_+ | 1364 | [M-C_27_H_44_NO_3_-C_6_H_10_O_4_-C_6_H_10_O_4_-2H_2_O+H]^+^ | M-Agly-DeOxHex-DeOxHex-2H_2_O |  | |
|  | 275.1126 | C_12_H_19_O_7_+ | 10024 | [M-C_27_H_44_NO_3_-C_6_H_10_O_5_-C_6_H_10_O_5_-H_2_O+H]^+^ | M-Agly-Hex-Hex-H_2_O |  | |
|  | 273.0967 | C_12_H_17_O_7_+ | 11740 | [M-C_27_H_44_NO_3_-C_6_H_10_O_4_-C_6_H_10_O_5_-2H_2_O+H]^+^ | M-Agly-DeOxHex-Hex-2H_2_O |  | |
|  | 271.0825 | C_12_H_15_O_7_+ | 390 | [M-C_27_H_44_NO_3_-C_6_H_10_O_4_-C_6_H_10_O_4_-3H_2_O+H]^+^ | M-Agly-DeOxHex-DeOxHex-3H_2_O |  | |
|  | 257.1023 | C_12_H_17_O_6_+ | 8020 | [M-C_27_H_44_NO_3_-C_6_H_10_O_5_-C_6_H_10_O_5_-2H_2_O+H]^+^ | M-Agly-Hex-Hex-2H_2_O |  | |
|  | 255.0868 | C_12_H_15_O_6_+ | 6444 | [M-C_27_H_44_NO_3_-C_6_H_10_O_4_-C_6_H_10_O_5_-3H_2_O+H]^+^ | M-Agly-DeOxHex-Hex-3H_2_O |  | |
|  | 239.0915 | C_12_H_15_O_5_+ | 8346 | [M-C_27_H_44_NO_3_-C_6_H_10_O_5_-C_6_H_10_O_5_-3H_2_O+H]^+^ | M-Agly-Hex-Hex-3H_2_O |  | |
|  | 237.0766 | C_12_H_13_O_5_+ | 2154 | [M-C_27_H_44_NO_3_-C_6_H_10_O_4_-C_6_H_10_O_5_-4H_2_O+H]^+^ | M-Agly-DeOxHex-Hex-4H_2_O |  | |
|  | 221.0807 | C_12_H_13_O_4_+ | 530 | [M-C_27_H_44_NO_3_-C_6_H_10_O_5_-C_6_H_10_O_5_-4H_2_O+H]^+^ | M-Agly-Hex-Hex-4H_2_O |  | |
|  | 163.0609 | C_6_H_11_O_5_+ | 20696 | [M-C_27_H_44_NO_3_-C_6_H_10_O_4_-C_6_H_10_O_4_-C_6_H_10_O_5_+H]^+^ | M-Agly-DeOxHex-DeOxHex-Hex | Hexose fragment | |
|  | 147.0657 | C_6_H_11_O_4_+ | 79068 | [M-C_27_H_44_NO_3_-C_6_H_10_O_4_-C_6_H_10_O_5_-C_6_H_10_O_5_+H]^+^ | M-Agly-DeOxHex-Hex-Hex | Deoxyhexose fragment | |
|  | 129.0550 | C_6_H_9_O_3_+ | 44014 | [M-C_27_H_44_NO_3_-C_6_H_10_O_4_-C_6_H_10_O_5_-C_6_H_10_O_5_-H_2_O+H]^+^ | M-Agly-DeOxHex-Hex-Hex-H_2_O |  | |
|  |  |  |  |  |  |  | |
| 10, D, 304, PRs/ARs | 1048.5679 | C_51_H_86_NO_21_+ | 602 | [M+H]^+^ | M | Putative molecular ion | |
|  | 902.5115 | C_45_H_76_NO_17_+ | 9226 | [M-C_6_H_10_O_4_]^+^ | M-DeOxHex | Triglycosidic steroid | |
|  | 740.4550 | C_39_H_66_NO_12_+ | 16756 | [M-C_6_H_10_O_4_-C_6_H_10_O_5_]^+^ | M-DeOxHex-DeOxHex | Diglycosidic steroid | |
|  | 594.3988 | C_33_H_56_NO_8_+ | 66026 | [M-C_6_H_10_O_4_-C_6_H_10_O_4_-C_6_H_10_O_5_]^+^ | M-DeOxHex-DeOxHex-Hex | Monoglycosidic steroid | |
|  | **432.3469** | C_27_H_46_NO_3_+ | 129674 | [M-C_6_H_10_O_4_-C_6_H_10_O_4_-C_6_H_10_O_5_-C_6_H_10_O_5_]^+^ | M-DeOxHex-DeOxHex-Hex-Hex | Aglycone | |
|  | 414.3366 | C_27_H_44_NO_2_+ | 4700 | [M-C_6_H_10_O_4_-C_6_H_10_O_4_-C_6_H_10_O_5_-C_6_H_10_O_5_-H_2_O]^+^ | M-DeOxHex-DeOxHex-Hex-Hex-H_2_O | Aglycone fragment | |
|  | 325.1119 | C_12_H_21_O_10_+ | 11878 | [M-C_27_H_46_NO_3_-C_6_H_10_O_4_-C_6_H_10_O_4_+H]^+^ | M-Agly-DeOxHex-DeOxHex | Disaccharide (Hex-Hex) | |
|  | 307.1012 | C_12_H_19_O_9_+ | 324 | [M-C_27_H_46_NO_3_-C_6_H_10_O_4_-C_6_H_10_O_4_-H_2_O+H]^+^ | M-Agly-DeOxHex-DeOxHex-H_2_O |  | |
|  | 289.0943 | C_12_H_17_O_8_+ | 866 | [M-C_27_H_46_NO_3_-C_6_H_10_O_4_-C_6_H_10_O_4_-2H_2_O+H]^+^ | M-Agly-DeOxHex-DeOxHex-2H_2_O |  | |
|  | 271.0815 | C_12_H_15_O_7_+ | 262 | [M-C_27_H_46_NO_3_-C_6_H_10_O_4_-C_6_H_10_O_4_-3H_2_O+H]^+^ | M-Agly-DeOxHex-DeOxHex-3H_2_O |  | |
|  | 309.1179 | C_12_H_21_O_9_+ | 30862 | [M-C_27_H_46_NO_3_-C_6_H_10_O_4_-C_6_H_10_O_5_+H]^+^ | M-Agly-DeOxHex-Hex | Disaccharide (DeOxHex-Hex) | |
|  | 291.1075 | C_12_H_19_O_8_+ | 2208 | [M-C_27_H_46_NO_3_-C_6_H_10_O_4_-C_6_H_10_O_5_-H_2_O+H]^+^ | M-Agly-DeOxHex-Hex-H_2_O |  | |
|  | 273.0969 | C_12_H_17_O_7_+ | 8766 | [M-C_27_H_46_NO_3_-C_6_H_10_O_4_-C_6_H_10_O_5_-2H_2_O+H]^+^ | M-Agly-DeOxHex-Hex-2H_2_O |  | |
|  | 255.0868 | C_12_H_15_O_6_+ | 4372 | [M-C_27_H_46_NO_3_-C_6_H_10_O_4_-C_6_H_10_O_5_-3H_2_O+H]^+^ | M-Agly-DeOxHex-Hex-3H_2_O |  | |
|  | 237.0771 | C_12_H_13_O_5_+ | 1312 | [M-C_27_H_46_NO_3_-C_6_H_10_O_4_-C_6_H_10_O_5_-4H_2_O+H]^+^ | M-Agly-DeOxHex-Hex-4H_2_O |  | |
|  | 163.0604 | C_6_H_11_O_5_+ | 17566 | [M-C_27_H_46_NO_3_-C_6_H_10_O_4_-C_6_H_10_O_4_-C_6_H_10_O_5_+H]^+^ | M-Agly-DeOxHex-DeOxHex-Hex | Hexose fragment | |
|  | 147.0656 | C_6_H_11_O_4_+ | 53974 | [M-C_27_H_46_NO_3_-C_6_H_10_O_4_-C_6_H_10_O_5_-C_6_H_10_O_5_+H]^+^ | M-Agly-DeOxHex-Hex-Hex | Deoxyhexose fragment | |
|  | 129.0550 | C_6_H_9_O_3_+ | 33578 | [M-C_27_H_46_NO_3_-C_6_H_10_O_4_-C_6_H_10_O_5_-C_6_H_10_O_5_-H_2_O+H]^+^ | M-Agly-DeOxHex-Hex-Hex-H_2_O |  | |
|  |  |  |  |  |  |  | |
| 11, I, 305.4, PRs | 1046.5572 | C_51_H_84_NO_21_+ | 576 | [M+H]^+^ | M | Putative molecular ion | |
|  | 884.5025 | C_45_H_74_NO_16_+ | 159908 | [M-C_6_H_10_O_5_]^+^ | M-Hex | Triglycosidic steroid | |
|  | 738.4444 | C_39_H_64_NO_12_+ | 122868 | [M-C_6_H_10_O_5_-C_6_H_10_O_4_]^+^ | M-Hex-DeOxHex | Diglycosidic steroid | |
|  | 592.3866 | C_33_H_54_NO_8_+ | 458112 | [M-C_6_H_10_O_5_-C_6_H_10_O_4_-C_6_H_10_O_4_]^+^ | M-Hex-DeOxHex-DeOxHex | Monoglycosidic steroid | |
|  | **430.3326** | C_27_H_44_NO_3_+ | 373452 | [M-C_6_H_10_O_5_-C_6_H_10_O_4_-C_6_H_10_O_4_-C_6_H_10_O_5_]^+^ | M-Hex-DeOxHex-DeOxHex-Hex | Aglycone | |
|  | 414.3363 | C_27_H_44_NO_2_+ | 5114 | [M-C_6_H_10_O_5_-C_6_H_10_O_4_-C_6_H_10_O_4_-C_6_H_10_O_5_-O]^+^ | M-Hex-DeOxHex-DeOxHex-Hex-Ox | Aglycone fragment | |
|  | 325.1132 | C_12_H_21_O_10_+ | 32072 | [M-C_27_H_44_NO_3_-C_6_H_10_O_4_-C_6_H_10_O_4_+H]^+^ | M-Agly-DeOxHex-DeOxHex | Disaccharide (Hex-Hex) | |
|  | 309.1180 | C_12_H_19_O_9_+ | 586 | [M-C_27_H_44_NO_3_-C_6_H_10_O_4_-C_6_H_10_O_4_-H_2_O+H]^+^ | M-Agly-DeOxHex-DeOxHex-H_2_O |  | |
|  | 307.1027 | C_12_H_17_O_8_+ | 1882 | [M-C_27_H_44_NO_3_-C_6_H_10_O_4_-C_6_H_10_O_4_-2H_2_O+H]^+^ | M-Agly-DeOxHex-DeOxHex-2H_2_O |  | |
|  | 293.1235 | C_12_H_15_O_7_+ | 516 | [M-C_27_H_44_NO_3_-C_6_H_10_O_4_-C_6_H_10_O_4_-3H_2_OH+H]^+^ | M-Agly-DeOxHex-DeOxHex-3H_2_O |  | |
|  | 291.1068 | C_12_H_13_O_6_+ | 318 | [M-C_27_H_44_NO_3_-C_6_H_10_O_4_-C_6_H_10_O_4_-4H_2_O+H]^+^ | M-Agly-DeOxHex-DeOxHex-4H_2_O |  | |
|  | 289.0919 | C_12_H_21_O_8_+ | 34360 | [M-C_27_H_44_NO_3_-C_6_H_10_O_5_-C_6_H_10_O_5_+H]^+^ | M-Agly-Hex-Hex | Disaccharide (DeOxHex-DeOxHex) | |
|  | 275.1123 | C_12_H_19_O_7_+ | 16002 | [M-C_27_H_44_NO_3_-C_6_H_10_O_5_-C_6_H_10_O_5_-H_2_O+H]^+^ | M-Agly-Hex-Hex-H_2_O |  | |
|  | 273.0972 | C_12_H_17_O_6_+ | 11410 | [M-C_27_H_44_NO_3_-C_6_H_10_O_5_-C_6_H_10_O_5_-2H_2_O+H]^+^ | M-Agly-Hex-Hex-2H_2_O |  | |
|  | 271.0818 | C_12_H_15_O_5_+ | 15406 | [M-C_27_H_44_NO_3_-C_6_H_10_O_5_-C_6_H_10_O_5_-3H_2_O+H]^+^ | M-Agly-Hex-Hex-3H_2_O |  | |
|  | 257.1021 | C_12_H_13_O_4_+ | 2598 | [M-C_27_H_44_NO_3_-C_6_H_10_O_5_-C_6_H_10_O_5_-4H_2_O+H]^+^ | M-Agly-Hex-Hex-4H_2_O |  | |
|  | 255.0871 | C_12_H_21_O_9_+ | 16786 | [M-C_27_H_44_NO_3_-C_6_H_10_O_4_-C_6_H_10_O_5_+H]^+^ | M-Agly-DeOxHex-Hex | Disaccharide (DeOxHex-Hex) | |
|  | 239.0919 | C_12_H_17_O_7_+ | 6328 | [M-C_27_H_44_NO_3_-C_6_H_10_O_4_-C_6_H_10_O_5_-2H_2_O+H]^+^ | M-Agly-DeOxHex-Hex-2H_2_O |  | |
|  | 237.0771 | C_12_H_15_O_6_+ | 3194 | [M-C_27_H_44_NO_3_-C_6_H_10_O_4_-C_6_H_10_O_5_-3H_2_O+H]^+^ | M-Agly-DeOxHex-Hex-3H_2_O |  | |
|  | 221.0808 | C_12_H_13_O_5_+ | 1016 | [M-C_27_H_44_NO_3_-C_6_H_10_O_4_-C_6_H_10_O_5_-4H_2_O+H]^+^ | M-Agly-DeOxHex-Hex-4H_2_O |  | |
|  | 163.0605 | C_6_H_11_O_5_+ | 36986 | [M-C_27_H_44_NO_3_-C_6_H_10_O_4_-C_6_H_10_O_4_-C_6_H_10_O_5_+H]^+^ | M-Agly-DeOxHex-DeOxHex-Hex | Hexose fragment | |
|  | 147.0659 | C_6_H_11_O_4_+ | 40994 | [M-C_27_H_44_NO_3_-C_6_H_10_O_4_-C_6_H_10_O_5_-C_6_H_10_O_5_+H]^+^ | M-Agly-DeOxHex-Hex-Hex | Deoxyhexose fragment | |
|  | 129.0551 | C_6_H_9_O_3_+ | 26804 | [M-C_27_H_44_NO_3_-C_6_H_10_O_4_-C_6_H_10_O_5_-C_6_H_10_O_5_-H_2_O+H]^+^ | M-Agly-DeOxHex-Hex-Hex-H_2_O |  | |
|  |  |  |  |  |  |  | |
| 12, C, 309.6, ARs/PRs | 1028.5461 | C_51_H_82_NO_20_+ | 415 | [M+H]^+^ | M | Putative molecular ion | |
|  | 882.4868 | C_45_H_72_NO_16_+ | 90624 | [M-C_6_H_10_O_4_]^+^ | M-DeOxHex | Triglycosidic steroid | |
|  | 736.4284 | C_39_H_62_NO_12_+ | 5252 | [M-C_6_H_10_O_4_-C_6_H_10_O_4_]^+^ | M-DeOxHex-DeOxHex | Diglycosidic steroid | |
|  | 720.4346 | C_39_H_62_NO_11_+ | 15738 | [M-C_6_H_10_O_4_-C_6_H_10_O_5_]^+^ | M-DeOxHex-Hex | Diglycosidic steroid | |
|  | **574.3762** | C_33_H_52_NO_7_ + | 276310 | [M-C_6_H_10_O_4_-C_6_H_10_O_4_-C_6_H_10_O_5_]^+^ | M-DeOxHex-DeOxHex-Hex | Monoglycosidic steroid | |
|  | 412.3220 | C_27_H_42_NO_2_+ | 559943 | [M-C_6_H_10_O_4_-C_6_H_10_O_4_-C_6_H_10_O_5_-C_6_H_10_O_5_]^+^ | M-DeOxHex-DeOxHex-Hex-Hex | Aglycone | |
|  | 309.1185 | C_12_H_21_O_9_+ | 34384 | [M-C_27_H_42_NO_2_-C_6_H_10_O_4_-C_6_H_10_O_5_+H]^+^ | M-Agly-DeOxHex-Hex | Disaccharide (DeOxHex-Hex) | |
|  | 291.1080 | C_12_H_19_O_8_+ | 2713 | [M-C_27_H_42_NO_2_-C_6_H_10_O_4_-C_6_H_10_O_5_-H_2_O+H]^+^ | M-Agly-DeOxHex-Hex-H_2_O |  | |
|  | 273.0970 | C_12_H_17_O_7_+ | 7705 | [M-C_27_H_42_NO_2_-C_6_H_10_O_4_-C_6_H_10_O_5_-2H_2_O+H]^+^ | M-Agly-DeOxHex-Hex-2H_2_O |  | |
|  | 255.0864 | C_12_H_15_O_6_+ | 4633 | [M-C_27_H_42_NO_2_-C_6_H_10_O_4_-C_6_H_10_O_5_-3H_2_O+H]^+^ | M-Agly-DeOxHex-Hex-3H_2_O |  | |
|  | 237.0773 | C_12_H_13_O_5_+ | 1021 | [M-C_27_H_42_NO_2_-C_6_H_10_O_4_-C_6_H_10_O_5_-4H_2_O+H]^+^ | M-Agly-DeOxHex-Hex-4H_2_O |  | |
|  | 163.0606 | C_6_H_11_O_5_+ | 8331 | [M-C_27_H_42_NO_2_-C_6_H_10_O_4_-C_6_H_10_O_4_-C_6_H_10_O_5_+H]^+^ | M-Agly-DeOxHex-DeOxHex-Hex | Hexose fragment | |
|  | 147.0659 | C_6_H_11_O_4_+ | 59512 | [M-C_27_H_42_NO_2_-C_6_H_10_O_4_-C_6_H_10_O_5_-C_6_H_10_O_5_+H]^+^ | M-Agly-DeOxHex-Hex-Hex | Deoxyhexose fragment | |
|  | 145.0502 | C_6_H_9_O_4_+ | 4805 | [M-C_27_H_42_NO_2_-C_6_H_10_O_4_-C_6_H_10_O_4_-C_6_H_10_O_5_-H_2_O+H]^+^ | M-Agly-DeOxHex-DeOxHex-Hex-H_2_O |  | |
|  | 129.0551 | C_6_H_9_O_3_+ | 33427 | [M-C_27_H_42_NO_2_-C_6_H_10_O_4_-C_6_H_10_O_5_-C_6_H_10_O_5_-H_2_O+H]^+^ | M-Agly-DeOxHex-Hex-Hex-H_2_O |  | |
|  | 127.0395 | C_6_H_7_O_3_+ | 2713 | [M-C_27_H_42_NO_2_-C_6_H_10_O_4_-C_6_H_10_O_4_-C_6_H_10_O_5_-2H_2_O+H]^+^ | M-Agly-DeOxHex-DeOxHex-Hex-2H_2_O |  | |
|  | 111.0446 | C_6_H_7_O_2_+ | 2380 | [M-C_27_H_42_NO_2_-C_6_H_10_O_4_-C_6_H_10_O_5_-C_6_H_10_O_5_-2H_2_O+H]^+^ | M-Agly-DeOxHex-Hex-Hex-2H_2_O |  | |
|  |  |  |  |  |  |  | |
| 13, E, 309, PRs | 1032.5352 | C_50_H_82_NO_21_+ | 244 | [M+H]^+^ | M | Putative molecular ion | |
|  | 900.4954 | C_45_H_74_NO_17_+ | 57154 | [M-C_5_H_8_O_4_]^+^ | M-Pent | Triglycosidic steroid | |
|  | 738.4433 | C_39_H_64_NO_12_+ | 55036 | [M-C_5_H_8_O_4_-C_6_H_10_O_5_]^+^ | M-Pent-Hex | Diglycosidic steroid | |
|  | 592.3856 | C_33_H_54_NO_8_+ | 367120 | [M-C_5_H_8_O_4_-C_6_H_10_O_5_-C_6_H_10_O_4_]^+^ | M-Pent-Hex-DeOxHex | Monoglycosidic steroid | |
|  | **430.3317** | C_27_H_44_NO_3_+ | 1458480 | [M-C_5_H_8_O_4_-C_6_H_10_O_5_-C_6_H_10_O_4_-C_6_H_10_O_5_]^+^ | M-Pent-Hex-DeOxHex-Hex | Aglycone | |
|  | 412.3204 | C_27_H_42_NO_2_+ | 29936 | [M-C_5_H_8_O_4_-C_6_H_10_O_5_-C_6_H_10_O_4_-C_6_H_10_O_5_-H_2_O ]^+^ | M-Pent-Hex-DeOxHex-Hex-H_2_O | Aglycone fragment | |
|  | 309.1177 | C_12_H_21_O_9_+ | 105402 | [M-C_27_H_44_NO_3_-C_5_H_8_O_4_-C_6_H_10_O_5_+H]^+^ | M-Agly-Pent-Hex | Disaccharide (DeOxHex-Hex) | |
|  | 291.1068 | C_12_H_19_O_8_+ | 6654 | [M-C_27_H_44_NO_3_-C_5_H_8_O_4_-C_6_H_10_O_5_-H_2_O+H]^+^ | M-Agly-Pent-Hex-H_2_O |  | |
|  | 273.0967 | C_12_H_17_O_7_+ | 23410 | [M-C_27_H_44_NO_3_-C_5_H_8_O_4_-C_6_H_10_O_5_-2H_2_O+H]^+^ | M-Agly-Pent-Hex-2H_2_O |  | |
|  | 255.0859 | C_12_H_15_O_6_+ | 15446 | [M-C_27_H_44_NO_3_-C_5_H_8_O_4_-C_6_H_10_O_5_-3H_2_O+H]^+^ | M-Agly-Pent-Hex-3H_2_O |  | |
|  | 237.0756 | C_12_H_13_O_5_+ | 2166 | [M-C_27_H_44_NO_3_-C_5_H_8_O_4_-C_6_H_10_O_5_-4H_2_O+H]^+^ | M-Agly-Pent-Hex-4H_2_O |  | |
|  | 147.0656 | C_6_H_11_O_4_+ | 149778 | [M-C_27_H_44_NO_3_-C_5_H_8_O_4_-C_6_H_10_O_5_-C_6_H_10_O_5_+H]^+^ | M-Agly-Pent-Hex-Hex | Deoxyhexose fragment | |
|  | 129.0547 | C_6_H_9_O_3_+ | 80052 | [M-C_27_H_44_NO_3_-C_5_H_8_O_4_-C_6_H_10_O_5_-C_6_H_10_O_5_-H_2_O+H]^+^ | M-Agly-Pent-Hex-Hex-H_2_O |  | |
|  | 111.0439 | C_6_H_7_O_2_+ | 4476 | [M-C_27_H_44_NO_3_-C_5_H_8_O_4_-C_6_H_10_O_5_-C_6_H_10_O_5_-2H_2_O+H]^+^ | M-Agly-Pent-Hex-Hex-2H_2_O |  | |
|  | 133.0496 | C_5_H_9_O_4_+ | 1288 | [M-C_27_H_44_NO_3_-C_6_H_10_O_5_ -C_6_H_10_O_5_-C_6_H_10_O_4_+H]^+^ | M-Agly-Hex-Hex-Hex | Pentose fragment | |
|  |  |  |  |  |  |  | |
| 14, C, 313.6, ARs/PRs | 1028.5477 | C_51_H_82_NO_20_+ | 73 | [M+H]^+^ | M | Putative molecular ion | |
|  | 866.4925 | C_45_H_72_NO_15_+ | 188997 | [M-C_6_H_10_O_5_]^+^ | M-Hex | Triglycosidic steroid | |
|  | 720.4345 | C_39_H_62_NO_11_+ | 17759 | [M-C_6_H_10_O_5_ - C_6_H_10_O_4_]^+^ | M-Hex-DeOxHex | Diglycosidic steroid | |
|  | **574.3762** | C_33_H_52_NO_7_+ | 260121 | [M-C_6_H_10_O_5_ - C_6_H_10_O_4_ - C_6_H_10_O_4_]^+^ | M-Hex-DeOxHex-DeOxHex | Monoglycosidic steroid | |
|  | 412.3218 | C_27_H_42_NO_2_+ | 78228 | [M-C_6_H_10_O_5_ - C_6_H_10_O_4_ - C_6_H_10_O_4_ - C_6_H_10_O_5_]^+^ | M-Hex-DeOxHex-DeOxHex-Hex | Aglycone | |
|  | 325.1112 | C_12_H_21_O_10_+ | 2154 | [M-C_27_H_42_NO_2_-C_6_H_10_O_4-_C_6_H_10_O_4_+H]^+^ | M-Agly-DeOxHex-DeOxHex | Disaccharide (Hex-Hex) | |
|  | 309.1182 | C_12_H_21_O_9_+ | 7497 | [M-C_27_H_42_NO_2_-C_6_H_10_O_4-_C_6_H_10_O_5_+H]^+^ | M-Agly-DeOxHex-Hex | Disaccharide (DeOxHex-Hex) | |
|  | 307.1013 | C_12_H_19_O_9_+ | 137 | [M-C_27_H_42_NO_2_-C_6_H_10_O_4-_C_6_H_10_O_4_-H_2_O+H]^+^ | M-Agly-DeOxHex-DeOxHex-H_2_O |  | |
|  | 291.1076 | C_12_H_19_O_8_+ | 633 | [M-C_27_H_42_NO_2_-C_6_H_10_O_4-_C_6_H_10_O_5_-H_2_O+H]^+^ | M-Agly-DeOxHex-Hex-H_2_O |  | |
|  | 293.1234 | C_12_H_21_O_8_+ | 15366 | [M-C_27_H_42_NO_2_-C_6_H_10_O_5-_C_6_H_10_O_5_+H]^+^ | M-Agly-Hex-Hex | Disaccharide (DeOxHex-DeOxHex) | |
|  | 289.0898 | C_12_H_17_O_8_+ | 261 | [M-C_27_H_42_NO_2_-C_6_H_10_O_4-_C_6_H_10_O_4_-2H_2_O+H]^+^ | M-Agly-DeOxHex-DeOxHex-2H_2_O |  | |
|  | 275.1128 | C_12_H_19_O_7_+ | 9134 | [M-C_27_H_42_NO_2_-C_6_H_10_O_5-_C_6_H_10_O_5_-H_2_O+H]^+^ | M-Agly-Hex-Hex-H_2_O |  | |
|  | 273.0971 | C_12_H_17_O_7_+ | 2335 | [M-C_27_H_42_NO_2_-C_6_H_10_O_4-_C_6_H_10_O_5_-2H_2_O+H]^+^ | M-Agly-DeOxHex-Hex-2H_2_O |  | |
|  | 271.0817 | C_12_H_15_O_7_+ | 104 | [M-C_27_H_42_NO_2_-C_6_H_10_O_4-_C_6_H_10_O_4_-3H_2_O+H]^+^ | M-Agly-DeOxHex-DeOxHex-3H_2_O |  | |
|  | 255.0873 | C_12_H_15_O_6_+ | 1258 | [M-C_27_H_42_NO_2_-C_6_H_10_O_4-_C_6_H_10_O_5_-3H_2_O+H]^+^ | M-Agly-DeOxHex-Hex-3H_2_O |  | |
|  | 257.1027 | C_12_H_17_O_6_+ | 5901 | [M-C_27_H_42_NO_2_-C_6_H_10_O_5-_C_6_H_10_O_5_-2H_2_O+H]^+^ | M-Agly-Hex-Hex-2H_2_O |  | |
|  | 239.0918 | C_12_H_15_O_5_+ | 7773 | [M-C_27_H_42_NO_2_-C_6_H_10_O_5-_C_6_H_10_O_5_-3H_2_O+H]^+^ | M-Agly-Hex-Hex-3H_2_O |  | |
|  | 237.0759 | C_12_H_13_O_5_+ | 648 | [M-C_27_H_42_NO_2_-C_6_H_10_O_4-_C_6_H_10_O_5_-4H_2_O+H]^+^ | M-Agly-DeOxHex-Hex-4H_2_O |  | |
|  | 221.0811 | C_12_H_13_O_4_+ | 449 | [M-C_27_H_42_NO_2_-C_6_H_10_O_5-_C_6_H_10_O_5_-4H_2_O+H]^+^ | M-Agly-Hex-Hex-4H_2_O |  | |
|  | 163.0608 | C_6_H_11_O_5_+ | 3102 | [M-C_27_H_42_NO_2-_C_6_H_10_O_4_-C_6_H_10_O_4-_C_6_H_10_O_5_+H]^+^ | M-Agly-DeOxHex-DeOxHex-Hex | Hexose fragment | |
|  | 145.0504 | C_6_H_9_O_4_+ | 1387 | [M-C_27_H_42_NO_2-_C_6_H_10_O_4_-C_6_H_10_O_4-_C_6_H_10_O_5_-H_2_O+H]^+^ | M-Agly-DeOxHex-DeOxHex-Hex-H_2_O |  | |
|  | 147.0659 | C_6_H_11_O_4_+ | 20736 | [M-C_27_H_42_NO_2-_C_6_H_10_O_4_-C_6_H_10_O_5-_C_6_H_10_O_5_+H]^+^ | M-Agly-DeOxHex-Hex-Hex | Deoxyhexose fragment | |
|  | 129.0552 | C_6_H_9_O_3_+ | 14658 | [M-C_27_H_42_NO_2-_C_6_H_10_O_4_-C_6_H_10_O_5-_C_6_H_10_O_5_-H_2_O+H]^+^ | M-Agly-DeOxHex-Hex-Hex-H_2_O |  | |
|  | 111.0446 | C_6_H_7_O_2_+ | 3270 | [M-C_27_H_42_NO_2-_C_6_H_10_O_4_-C_6_H_10_O_5-_C_6_H_10_O_5_-2H_2_O+H]^+^ | M-Agly-DeOxHex-Hex-Hex-2H_2_O |  | |
|  |  |  |  |  |  |  | |
| 15, H, 313.9, PRs | 1050.5435 | C_50_H_84_NO_22_+ | 448 | [M+H]^+^ | M | Putative molecular ion | |
|  | 918.5088 | C_45_H_76_NO_18_+ | 11632 | [M-C_5_H_8_O_4_]^+^ | M-Pent | Triglycosidic steroid | |
|  | 756.4541 | C_39_H_66_NO_13_+ | 2128 | [M-C_5_H_8_O_4_-C_6_H_10_O_5_]^+^ | M-Pent-Hex | Diglycosidic steroid | |
|  | 594.4021 | C_33_H_56_NO_8_+ | 298158 | [M-C_5_H_8_O_4_-C_6_H_10_O_5_-C_6_H_10_O_5_]^+^ | M-Pent-Hex-Hex | Monoglycosidic steroid | |
|  | **432.3479** | C_27_H_46_NO_3_+ | 466752 | [M-C_5_H_8_O_4_-C_6_H_10_O_5_-C_6_H_10_O_5_-C_6_H_10_O_5_]^+^ | M-Pent-Hex-Hex-Hex | Aglycone | |
|  | 416.3516 | C_27_H_46_NO_2_+ | 6612 | [M-C_5_H_8_O_4_-C_6_H_10_O_5_-C_6_H_10_O_5_-C_6_H_10_O_5_-O]^+^ | M-Pent-Hex-Hex-Hex-Ox | Aglycone fragment | |
|  | 325.1126 | C_12_H_21_O_10_+ | 80688 | [M-C_27_H_46_NO_3_-C_5_H_8_O_4_-C_6_H_10_O_5_+H]^+^ | M-Agly-Pent-Hex | Disaccharide (Hex-Hex) | |
|  | 309.1176 | C_12_H_21_O_9_+ | 27698 | [M-C_27_H_46_NO_3_-C_5_H_8_O_4_-C_6_H_10_O_5_-O+H]^+^ | M-Agly-Pent-Hex-Ox | Disaccharide (DeOxHex-Hex) | |
|  | 289.0918 | C_12_H_17_O_8_+ | 6556 | [M-C_27_H_46_NO_3_-C_5_H_8_O_4_-C_6_H_10_O_5_-2H_2_O+H]^+^ | M-Agly-Pent-Hex-2H_2_O |  | |
|  | 271.0823 | C_12_H_15_O_7_+ | 1326 | [M-C_27_H_46_NO_3_-C_5_H_8_O_4_-C_6_H_10_O_5_-3H_2_O+H]^+^ | M-Agly-Pent-Hex-3H_2_O |  | |
|  | 253.0697 | C_12_H_13_O_6_+ | 660 | [M-C_27_H_46_NO_3_-C_5_H_8_O_4_-C_6_H_10_O_5_-4H_2_O+H]^+^ | M-Agly-Pent-Hex-4H_2_O |  | |
|  | 295.1022 | C_11_H_19_O_9_+ | 46406 | [M-C_27_H_46_NO_3_-C_6_H_10_O_5_-C_6_H_10_O_5_+H]^+^ | M-Agly-Hex-Hex |  | |
|  | 277.0908 | C_11_H_17_O_8_+ | 1754 | [M-C_27_H_46_NO_3_-C_6_H_10_O_5_-C_6_H_10_O_5_-H_2_O+H]^+^ | M-Agly-Hex-Hex-H_2_O |  | |
|  | 259.0811 | C_11_H_15_O_7_+ | 5138 | [M-C_27_H_46_NO_3_-C_6_H_10_O_5_-C_6_H_10_O_5_-2H_2_O+H]^+^ | M-Agly-Hex-Hex-2H_2_O |  | |
|  | 241.0698 | C_11_H_13_O_6_+ | 2528 | [M-C_27_H_46_NO_3_-C_6_H_10_O_5_-C_6_H_10_O_5_-3H_2_O+H]^+^ | M-Agly-Hex-Hex-3H_2_O |  | |
|  | 223.0603 | C_11_H_11_O_5_+ | 952 | [M-C_27_H_46_NO_3_-C_6_H_10_O_5_-C_6_H_10_O_5_-4H_2_O+H]^+^ | M-Agly-Hex-Hex-4H_2_O |  | |
|  | 163.0607 | C_6_H_11_O_5_+ | 86856 | [M-C_27_H_46_NO_3_-C_5_H_8_O_4_-C_6_H_10_O_5_-C_6_H_10_O_5_+H]^+^ | M-Agly-Pent-Hex-Hex | Hexose fragment | |
|  | 145.0500 | C_6_H_9_O_4_+ | 51716 | [M-C_27_H_46_NO_3_-C_5_H_8_O_4_-C_6_H_10_O_5_-C_6_H_10_O_5_-H_2_O+H]^+^ | M-Agly-Pent-Hex-Hex-H_2_O |  | |
|  | 133.0502 | C_5_H_9_O_4_+ | 6240 | [M-C_27_H_46_NO_3_-C_6_H_10_O_5_ -C_6_H_10_O_5_-C_6_H_10_O_5_+H]^+^ | M-Agly-Hex-Hex-Hex | Pentose fragment | |
|  | 127.0392 | C_6_H_7_O_3_+ | 11454 | [M-C_27_H_46_NO_3_-C_5_H_8_O_4_-C_6_H_10_O_5_-C_6_H_10_O_5_-2H_2_O+H]^+^ | M-Agly-Pent-Hex-Hex-2H_2_O |  | |
|  | 109.0285 | C_6_H_5_O_2_+ | 1248 | [M-C_27_H_46_NO_3_-C_5_H_8_O_4_-C_6_H_10_O_5_-C_6_H_10_O_5_-3H_2_O+H]^+^ | M-Agly-Pent-Hex-Hex-3H_2_O |  | |
|  |  |  |  |  |  |  | |
| 16, I, 316, PRs/ARs | 884.5004 | C_45_H_74_NO_16_+ | 136960 | [M+H]^+^ | M | Putative molecular ion | |
|  | 738.4432 | C_39_H_64_NO_12_+ | 84012 | [M-C_6_H_10_O_4_]^+^ | M-DeOxHex | Diglycosidic steroid | |
|  | 592.3851 | C_33_H_54_NO_8_+ | 332666 | [M-C_6_H_10_O_4_-C_6_H_10_O_4_]^+^ | M-DeOxHex-DeOxHex | Monoglycosidic steroid | |
|  | **430.3314** | C_27_H_44_NO_3_+ | 201148 | [M-C_6_H_10_O_4_-C_6_H_10_O_4_-C_6_H_10_O_5_]^+^ | M-DeOxHex-DeOxHex-Hex | Aglycone | |
|  | 414.3351 | C_27_H_44_NO_2_+ | 8472 | [M-C_6_H_10_O_4_-C_6_H_10_O_4_-C_6_H_10_O_5_-O]^+^ | M-DeOxHex-DeOxHex-Hex-Ox | Aglycone fragment | |
|  | 293.1221 | C_12_H_21_O_8_+ | 35330 | [M-C_27_H_44_NO_3_-C_6_H_10_O_5_+H]^+^ | M-Agly-Hex | Disaccharide (DeOxHex-DeOxHex) | |
|  | 275.1125 | C_12_H_19_O_7_+ | 17560 | [M-C_27_H_44_NO_3_-C_6_H_10_O_5_-H_2_O+H]^+^ | M-Agly-Hex-H_2_O |  | |
|  | 257.1014 | C_12_H_17_O_6_+ | 11262 | [M-C_27_H_44_NO_3_-C_6_H_10_O_5_-2H_2_O+H]^+^ | M-Agly-Hex-2H_2_O |  | |
|  | 239.0914 | C_12_H_15_O_5_+ | 17630 | [M-C_27_H_44_NO_3_-C_6_H_10_O_5_-3H_2_O+H]^+^ | M-Agly-Hex-3H_2_O |  | |
|  | 147.0654 | C_6_H_11_O_4_+ | 38268 | [M-C_27_H_44_NO_3_-C_6_H_10_O_4_-C_6_H_10_O_5_+H]^+^ | M-Agly-DeOxHex-Hex | Deoxyhexose fragment | |
|  | 129.0547 | C_6_H_9_O_3_+ | 23414 | [M-C_27_H_44_NO_3_-C_6_H_10_O_4_-C_6_H_10_O_5_-H_2_O+H]^+^ | M-Agly-DeOxHex-Hex-H_2_O |  | |
|  | 111.0437 | C_6_H_7_O_2_+ | 6096 | [M-C_27_H_44_NO_3_-C_6_H_10_O_4_-C_6_H_10_O_5_-2H_2_O+H]^+^ | M-Agly-DeOxHex-Hex-2H_2_O |  | |
|  |  |  |  |  |  |  | |
| 17, D, 320, PRs | 886.5083 | C_45_H_76_NO_16_+ | 1130 | [M+H]^+^ | M | Putative molecular ion | |
|  | 740.4528 | C_39_H_66_NO_12_+ | 69581 | [M-C_6_H_10_O_4_]^+^ | M-DeOxHex | Diglycosidic steroid | |
|  | 594.4002 | C_33_H_56_NO_8_+ | 2475 | [M-C_6_H_10_O_4_-C_6_H_10_O_4_]^+^ | M-DeOxHex-DeOxHex | Monoglycosidic steroid | |
|  | **432.3482** | C_27_H_46_NO_3_+ | 5786 | [M-C_6_H_10_O_4_-C_6_H_10_O_4_-C_6_H_10_O_5_]^+^ | M-DeOxHex-DeOxHex-Hex | Aglycone | |
|  | 414.3380 | C_27_H_44_NO_2_+ | 73061 | [M-C_6_H_10_O_4_-C_6_H_10_O_4_-C_6_H_10_O_5_-H_2_O]^+^ | M-DeOxHex-DeOxHex-Hex-H_2_O | Aglycone fragment | |
|  | 309.1185 | C_12_H_21_O_9_+ | 8185 | [M-C_27_H_46_NO_3_-C_6_H_10_O_4_+H]^+^ | M-Agly-DeOxHex | Disaccharide (DeOxHex-Hex) | |
|  | 291.1079 | C_12_H_19_O_8_+ | 817 | [M-C_27_H_46_NO_3_-C_6_H_10_O_4_-H_2_O+H]^+^ | M-Agly-DeOxHex-H_2_O |  | |
|  | 273.0973 | C_12_H_17_O_7_+ | 1896 | [M-C_27_H_46_NO_3_-C_6_H_10_O_4_-2H_2_O+H]^+^ | M-Agly-DeOxHex-2H_2_O |  | |
|  | 255.0868 | C_12_H_15_O_6_+ | 1191 | [M-C_27_H_46_NO_3_-C_6_H_10_O_4_-3H_2_O+H]^+^ | M-Agly-DeOxHex-3H_2_O |  | |
|  | 237.0760 | C_12_H_13_O_5_+ | 375 | [M-C_27_H_46_NO_3_-C_6_H_10_O_4_-4H_2_O+H]^+^ | M-Agly-DeOxHex-4H_2_O |  | |
|  | 147.0661 | C_6_H_11_O_4_+ | 13681 | [M-C_27_H_46_NO_3_-C_6_H_10_O_4_-C_6_H_10_O_5_+H]^+^ | M-Agly-DeOxHex-Hex | Deoxyhexose fragment | |
|  | 129.0553 | C_6_H_9_O_3_+ | 7251 | [M-C_27_H_46_NO_3_-C_6_H_10_O_4_-C_6_H_10_O_5_-H_2_O+H]^+^ | M-Agly-DeOxHex-Hex-H_2_O |  | |
|  | 111.0451 | C_6_H_7_O_2_+ | 853 | [M-C_27_H_46_NO_3_-C_6_H_10_O_4_-C_6_H_10_O_5_-2H_2_O+H]^+^ | M-Agly-DeOxHex-Hex-2H_2_O |  | |
|  |  |  |  |  |  |  | |
| 18, B, 324, PRs/ARs | 868.5114 | C_45_H_74_NO_15_+ | 10960 | [M+H]^+^ | M | Putative molecular ion | |
|  | 722.4498 | C_39_H_64_NO_11_+ | 2237 | [M-C_6_H_10_O_4_]^+^ | M-DeOxHex | Diglycosidic steroid | |
|  | 576.3935 | C_33_H_54_NO_7_+ | 24844 | [M-C_6_H_10_O_4_-C_6_H_10_O_4_]^+^ | M-DeOxHex-DeOxHex | Monoglycosidic steroid | |
|  | **414.3386** | C_27_H_44_NO_2_+ | 18599 | [M-C_6_H_10_O_4_-C_6_H_10_O_4_-C_6_H_10_O_5_]^+^ | M-DeOxHex-DeOxHex-Hex | Aglycone | |
|  | 309.1201 | C_12_H_21_O_9_+ | 2562 | [M-C_27_H_44_NO_2_-C_6_H_10_O_4_+H]^+^ | M-Agly-DeOxHex | Disaccharide (DeOxHex-Hex) | |
|  | 147.0664 | C_6_H_11_O_4_+ | 5011 | [M-C_27_H_44_NO_2_-C_6_H_10_O_4_-C_6_H_10_O_5_+H]^+^ | M-Agly-DeOxHex-Hex | Deoxyhexose fragment | |
|  | 129.0557 | C_6_H_9_O_3_+ | 3845 | [M-C_27_H_44_NO_2_-C_6_H_10_O_4_-C_6_H_10_O_5_-H_2_O+H]^+^ | M-Agly-DeOxHex-Hex-H_2_O |  | |
|  |  |  |  |  |  |  | |
| 19, B, 336.1, PRs/ARs | 1046.5539 | C_51_H_84_NO_21_+ | 487 | [M+H]^+^ | M | Putative molecular ion | |
|  | 884.5077 | C_45_H_74_NO_16_+ | 277 | [M-C_6_H_10_O_5_]^+^ | M-Hex | Triglycosidic steroid | |
|  | 722.4496 | C_39_H_64_NO_11_+ | 7778 | [M-C_6_H_10_O_5_-C_6_H_10_O_5_]^+^ | M-Hex-Hex | Diglycosidic steroid | |
|  | 576.3916 | C_33_H_54_NO_7_+ | 67593 | [M-C_6_H_10_O_5_-C_6_H_10_O_5-_C_6_H_10_O_4_]^+^ | M-Hex-Hex-DeOxHex | Monoglycosidic steroid | |
|  | **414.3381** | C_27_H_44_NO_2_+ | 232700 | [M-C_6_H_10_O_5_-C_6_H_10_O_5_-C_6_H_10_O_4_-C_6_H_10_O_5_]^+^ | M-Hex-Hex-DeOxHex-Hex | Aglycone | |
|  | 325.1132 | C_12_H_21_O_10_+ | 12903 | [M-C_27_H_44_NO_2_-C_6_H_10_O_4_-C_6_H_10_O_5_+H]^+^ | M-Agly-DeOxHex-Hex | Disaccharide (Hex-Hex) | |
|  | 307.1040 | C_12_H_19_O_9_+ | 114 | [M-C_27_H_44_NO_2_-C_6_H_10_O_4_-C_6_H_10_O_5_-H_2_O+H]^+^ | M-Agly-DeOxHex-Hex-H_2_O |  | |
|  | 289.0926 | C_12_H_17_O_8_+ | 1165 | [M-C_27_H_44_NO_2_-C_6_H_10_O_4_-C_6_H_10_O_5_-2H_2_O+H]^+^ | M-Agly-DeOxHex-Hex-2H_2_O |  | |
|  | 271.0809 | C_12_H_15_O_7_+ | 354 | [M-C_27_H_44_NO_2_-C_6_H_10_O_4_-C_6_H_10_O_5_-3H_2_O+H]^+^ | M-Agly-DeOxHex-Hex-3H_2_O |  | |
|  | 253.0707 | C_12_H_13_O_6_+ | 75 | [M-C_27_H_44_NO_2_-C_6_H_10_O_4_-C_6_H_10_O_5_-4H_2_O+H]^+^ | M-Agly-DeOxHex-Hex-4H_2_O |  | |
|  | 309.1187 | C_12_H_21_O_9_+ | 65557 | [M-C_27_H_44_NO_2_-C_6_H_10_O_5_-C_6_H_10_O_5_+H]^+^ | M-Agly-Hex-Hex | Disaccharide (DeOxHex-Hex) | |
|  | 291.1076 | C_12_H_19_O_8_+ | 2825 | [M-C_27_H_44_NO_2_-C_6_H_10_O_5_-C_6_H_10_O_5_-H_2_O+H]^+^ | M-Agly-Hex-Hex-H_2_O |  | |
|  | 273.0972 | C_12_H_17_O_7_+ | 11516 | [M-C_27_H_44_NO_2_-C_6_H_10_O_5_-C_6_H_10_O_5_-2H_2_O+H]^+^ | M-Agly-Hex-Hex-2H_2_O |  | |
|  | 255.0864 | C_12_H_15_O_6_+ | 4165 | [M-C_27_H_44_NO_2_-C_6_H_10_O_5_-C_6_H_10_O_5_-3H_2_O+H]^+^ | M-Agly-Hex-Hex-3H_2_O |  | |
|  | 237.0758 | C_12_H_13_O_5_+ | 1145 | [M-C_27_H_44_NO_2_-C_6_H_10_O_5_-C_6_H_10_O_5_-4H_2_O+H]^+^ | M-Agly-Hex-Hex-4H_2_O |  | |
|  | 163.0607 | C_6_H_11_O_5_+ | 13286 | [M-C_27_H_46_NO_3_-C_6_H_10_O_5_-C_6_H_10_O_5_-C_6_H_10_O_4_+H]^+^ | M-Agly-Hex-Hex-DeOxHex | Hexose fragment | |
|  | 145.0502 | C_6_H_9_O_4_+ | 7776 | [M-C_27_H_46_NO_3_-C_6_H_10_O_5_-C_6_H_10_O_5_-C_6_H_10_O_4_-H_2_O+H]^+^ | M-Agly-Hex-Hex-DeOxHex-H_2_O |  | |
|  | 127.0395 | C_6_H_7_O_3_+ | 2824 | [M-C_27_H_46_NO_3_-C_6_H_10_O_5_-C_6_H_10_O_5_-C_6_H_10_O_4_-2H_2_O+H]^+^ | M-Agly-Hex-Hex-DeOxHex-2H_2_O |  | |
|  | 147.0661 | C_6_H_11_O_4_+ | 34067 | [M-C_27_H_46_NO_3_-C_6_H_10_O_5_-C_6_H_10_O_5_-C_6_H_10_O_5_+H]^+^ | M-Agly-Hex-Hex-Hex | Deoxyhexose fragment | |
|  | 129.0552 | C_6_H_9_O_3_+ | 17246 | [M-C_27_H_46_NO_3_-C_6_H_10_O_5_-C_6_H_10_O_5_-C_6_H_10_O_5_-H_2_O+H]^+^ | M-Agly-Hex-Hex-Hex-H_2_O |  | |
|  | 111.0445 | C_6_H_7_O_2_+ | 1577 | [M-C_27_H_46_NO_3_-C_6_H_10_O_5_-C_6_H_10_O_5_-C_6_H_10_O_5_-2H_2_O+H]^+^ | M-Agly-Hex-Hex-Hex-2H_2_O |  | |
|  |  |  |  |  |  |  | |
| 20, B, 343.8, PRs/ARs | 1046.5564 | C_51_H_84_NO_21_+ | 30 | [M+H]^+^ | M | Putative molecular ion | |
|  | 884.5031 | C_45_H_74_NO_16_+ | 606282 | [M-C_6_H_10_O_5_]^+^ | M-Hex | Triglycosidic steroid | |
|  | 738.4441 | C_39_H_64_NO_12_+ | 25807 | [M-C_6_H_10_O_4_-C_6_H_10_O_5_]^+^ | M-DeOxHex-Hex | Diglycosidic steroid | |
|  | 722.4504 | C_39_H_64_NO_11_+ | 101238 | [M-C_6_H_10_O_5_-C_6_H_10_O_5_]^+^ | M-Hex-Hex | Diglycosidic steroid | |
|  | 576.3917 | C_33_H_54_NO_7_+ | 1459421 | [M-C_6_H_10_O_5_-C_6_H_10_O_5_-C_6_H_10_O_4_]^+^ | M-Hex-Hex-DeOxHex | Monoglycosidic steroid | |
|  | **414.3377** | C_27_H_44_NO_2_+ | 2808193 | [M-C_6_H_10_O_5_-C_6_H_10_O_5_-C_6_H_10_O_4_-C_6_H_10_O_5_]^+^ | M-Hex-Hex-DeOxHex-Hex | Aglycone | |
|  | 325.1127 | C_12_H_21_O_10_+ | 3088 | [M-C_27_H_44_NO_2_-C_6_H_10_O_4_-C_6_H_10_O_5_+H]^+^ | M-Agly-DeoOxHex-Hex | Disaccharide (Hex-Hex) | |
|  | 307.1046 | C_12_H_19_O_9_+ | 188 | [M-C_27_H_44_NO_2_-C_6_H_10_O_4_-C_6_H_10_O_5_-H_2_O+H]^+^ | M-Agly-DeOxHex-Hex-H_2_O |  | |
|  | 289.0905 | C_12_H_17_O_8_+ | 430 | [M-C_27_H_44_NO_2_-C_6_H_10_O_4_-C_6_H_10_O_5_-2H_2_O+H]^+^ | M-Agly-DeOxHex-Hex-2H_2_O |  | |
|  | 309.1189 | C_12_H_21_O_9_+ | 150362 | [M-C_27_H_44_NO_2_-C_6_H_10_O_5_-C_6_H_10_O_5_+H]^+^ | M-Agly-Hex-Hex | Disaccharide (DeOxHex-Hex) | |
|  | 291.1080 | C_12_H_19_O_8_+ | 9518 | [M-C_27_H_44_NO_2_-C_6_H_10_O_5_-C_6_H_10_O_5_-H_2_O+H]^+^ | M-Agly-Hex-Hex-H_2_O |  | |
|  | 273.0976 | C_12_H_17_O_7_+ | 29649 | [M-C_27_H_44_NO_2_-C_6_H_10_O_5_-C_6_H_10_O_5_-2H_2_O+H]^+^ | M-Agly-Hex-Hex-2H_2_O |  | |
|  | 255.0870 | C_12_H_15_O_6_+ | 19364 | [M-C_27_H_44_NO_2_-C_6_H_10_O_5_-C_6_H_10_O_5_-3H_2_O+H]^+^ | M-Agly-Hex-Hex-3H_2_O |  | |
|  | 237.0764 | C_12_H_13_O_5_+ | 2999 | [M-C_27_H_44_NO_2_-C_6_H_10_O_5_-C_6_H_10_O_5_-4H_2_O+H]^+^ | M-Agly-Hex-Hex-4H_2_O |  | |
|  | 163.0612 | C_6_H_11_O_5_+ | 23405 | [M-C_27_H_44_NO_2_-C_6_H_10_O_4_-C_6_H_10_O_5_-C_6_H_10_O_5_+H]^+^ | M-Agly-DeOxHex-Hex-Hex | Hexose fragment | |
|  | 145.0502 | C_6_H_9_O_4_+ | 18484 | [M-C_27_H_44_NO_2_-C_6_H_10_O_4_-C_6_H_10_O_5_-C_6_H_10_O_5_-H_2_O+H]^+^ | M-Agly-DeOxHex-Hex-Hex-H_2_O |  | |
|  | 127.0395 | C_6_H_7_O_3_+ | 10795 | [M-C_27_H_44_NO_2_-C_6_H_10_O_4_-C_6_H_10_O_5_-C_6_H_10_O_5_-2H_2_O+H]^+^ | M-Agly-DeOxHex-Hex-Hex-2H_2_O |  | |
|  | 109.0282 | C_6_H_5_O_2_+ | 1329 | [M-C_27_H_44_NO_2_-C_6_H_10_O_4_-C_6_H_10_O_5_-C_6_H_10_O_5_-3H_2_O+H]^+^ | M-Agly-DeOxHex-Hex-Hex-3H_2_O |  | |
|  | 147.0663 | C_6_H_11_O_4_+ | 232410 | [M-C_27_H_44_NO_2_-C_6_H_10_O_5_-C_6_H_10_O_5_-C_6_H_10_O_5_+H]^+^ | M-Agly-Hex-Hex-Hex | Deoxyhexose fragment | |
|  | 129.0554 | C_6_H_9_O_3_+ | 108762 | [M-C_27_H_44_NO_2_-C_6_H_10_O_5_-C_6_H_10_O_5_-C_6_H_10_O_5_-H_2_O+H]^+^ | M-Agly-Hex-Hex-Hex-H_2_O |  | |
|  | 111.0444 | C_6_H_7_O_2_+ | 8434 | [M-C_27_H_44_NO_2_-C_6_H_10_O_5_-C_6_H_10_O_5_-C_6_H_10_O_5_-2H_2_O+H]^+^ | M-Agly-Hex-Hex-Hex-2H_2_O |  | |
|  |  |  |  |  |  |  | |
| 21, B, 352.5, PRs/ARs | 1030.5561 | C_51_H_84_NO_20_+ | 232 | [M+H]^+^ | M | Putative molecular ion | |
|  | 884.4999 | C_45_H_74_NO_16_+ | 296069 | [M-C_6_H_10_O_4_]^+^ | M-DeOxHex | Triglycosidic steroid | |
|  | 868.5059 | C_45_H_74_NO_15_+ | 5170847 | [M-C_6_H_10_O_5_]^+^ | M-Hex | Triglycosidic steroid | |
|  | 738.4427 | C_39_H_64_NO_12_+ | 11348 | [M-C_6_H_10_O_4_-C_6_H_10_O_4_]^+^ | M-DeOxHex-DeOxHex | Diglycosidic steroid | |
|  | 722.4479 | C_39_H_64_NO_11_+ | 168219 | [M-C_6_H_10_O_4_-C_6_H_10_O_5_]^+^ | M-DeOxHex-Hex | Diglycosidic steroid | |
|  | 576.3902 | C_33_H_54_NO_7_+ | 3665672 | [M-C_6_H_10_O_4_-C_6_H_10_O_4_-C_6_H_10_O_5_]^+^ | M-DeOxHex-DeOxHex-Hex | Monoglycosidic steroid | |
|  | **414.3368** | C_27_H_44_NO_2_+ | 1384264 | [M-C_6_H_10_O_4_-C_6_H_10_O_4_-C_6_H_10_O_5_-C_6_H_10_O_5_]^+^ | M-DeOxHex-DeOxHex-Hex-Hex | Aglycone | |
|  | 325.1121 | C_12_H_21_O_10_+ | 1449 | [M-C_27_H_44_NO_2_-C_6_H_10_O_4_-C_6_H_10_O_4_+H]^+^ | M-Agly-DeOxHex-DeOxHex | Disaccharide (Hex-Hex) | |
|  | 309.1179 | C_12_H_21_O_9_+ | 55255 | [M-C_27_H_44_NO_2_-C_6_H_10_O_4_-C_6_H_10_O_5_+H]^+^ | M-Agly-DeOxHex-Hex | Disaccharide (DeOxHex-Hex) | |
|  | 291.1079 | C_12_H_19_O_8_+ | 3599 | [M-C_27_H_44_NO_2_-C_6_H_10_O_4_-C_6_H_10_O_5_-H_2_O+H]^+^ | M-Agly-DeOxHex-Hex-H_2_O |  | |
|  | 273.0963 | C_12_H_17_O_7_+ | 18439 | [M-C_27_H_44_NO_2_-C_6_H_10_O_4_-C_6_H_10_O_5_-2H_2_O+H]^+^ | M-Agly-DeOxHex-Hex-2H_2_O |  | |
|  | 255.0861 | C_12_H_15_O_6_+ | 11198 | [M-C_27_H_44_NO_2_-C_6_H_10_O_4_-C_6_H_10_O_5_-3H_2_O+H]^+^ | M-Agly-DeOxHex-Hex-3H_2_O |  | |
|  | 237.0761 | C_12_H_13_O_5_+ | 3562 | [M-C_27_H_44_NO_2_-C_6_H_10_O_4_-C_6_H_10_O_5_-4H_2_O+H]^+^ | M-Agly-DeOxHex-Hex-4H_2_O |  | |
|  | 221.0812 | C_12_H_13_O_4_+ | 3828 | [M-C_27_H_44_NO_2_-C_6_H_10_O_5_-C_6_H_10_O_5_-4H_2_O+H]^+^ | M-Agly-Hex-Hex-4H_2_O |  | |
|  | 163.0604 | C_6_H_11_O_5_+ | 16593 | [M-C_27_H_44_NO_2_-C_6_H_10_O_5_-C_6_H_10_O_5_-C_6_H_10_O_4_+H]^+^ | M-Agly-Hex-Hex-DeOxHex | Hexose fragment | |
|  | 147.0657 | C_6_H_11_O_4_+ | 155076 | [M-C_27_H_44_NO_2_-C_6_H_10_O_5_-C_6_H_10_O_4_-C_6_H_10_O_4_+H]^+^ | M-Agly-Hex-DeOxHex-DeOxHex | Deoxyhexose fragment | |
|  | 129.0549 | C_6_H_9_O_3_+ | 102615 | [M-C_27_H_44_NO_2_-C_6_H_10_O_5_-C_6_H_10_O_4_-C_6_H_10_O_4_-H_2_O+H]^+^ | M-Agly-Hex-DeOxHex-DeOxHex-H_2_O |  | |
|  | 111.0442 | C_6_H_7_O_2_+ | 27838 | [M-C_27_H_44_NO_2_-C_6_H_10_O_5_-C_6_H_10_O_4_-C_6_H_10_O_4_-2H_2_O+H]^+^ | M-Agly-Hex-DeOxHex-DeOxHex-2H_2_O |  | |
|  |  |  |  |  |  |  | |
| 22, B, 361.1, PRs/ARs | 1032.5398 | C_50_H_82_NO_21_+ | 905 | [M+H]^+^ | M | Putative molecular ion | |
|  | 884.5004 | C_45_H_74_NO_16_+ | 1057 | [M-C_5_H_8_O_5_]^+^ | M-Pent | Triglycosidic steroid | |
|  | 722.4500 | C_39_H_64_NO_11_+ | 7304 | [M-C_5_H_8_O_5_-C_6_H_10_O_5_]^+^ | M-Pent-Hexose | Diglycosidic steroid | |
|  | **576.3914** | C_33_H_54_NO_7_+ | 347680 | [M-C_5_H_8_O_5_-C_6_H_10_O_5_-C_6_H_10_O_4_]^+^ | M-Pent-Hex-DeOxHex | Monoglycosidic steroid | |
|  | 414.3371 | C_27_H_44_NO_2_+ | 162586 | [M-C_5_H_8_O_5_-C_6_H_10_O_4_-C_6_H_10_O_5_ -C_6_H_10_O_5_]^+^ | M-Pent-Hex-DeOxHex-Hex | Aglycone | |
|  | 325.1129 | C_12_H_21_O_10_+ | 58342 | [M-C_27_H_44_NO_2_-C_5_H_8_O_4_-C_6_H_10_O_5_+H]^+^ | M-Agly-Pent-Hex | Disaccharide (Hex-Hex) | |
|  | 295.1026 | C_11_H_19_O_9_+ | 104966 | [M-C_27_H_44_NO_2_-C_6_H_10_O_5_-C_6_H_10_O_5_+H]^+^ | M-Agly-Hex-Hex |  | |
|  | 259.0813 | C_11_H_15_O_7_+ | 11665 | [M-C_27_H_44_NO_2_-C_6_H_10_O_5_-C_6_H_10_O_5_-2H_2_O+H]^+^ | M-Agly-Hex-Hex-2H_2_O |  | |
|  | 241.0707 | C_11_H_13_O_6_+ | 5343 | [M-C_27_H_44_NO_2_-C_6_H_10_O_5_-C_6_H_10_O_5_-3H_2_O+H]^+^ | M-Agly-Hex-Hex-3H_2_O |  | |
|  | 223.0613 | C_11_H_11_O_5_+ | 2321 | [M-C_27_H_44_NO_2_-C_6_H_10_O_5_-C_6_H_10_O_5_-4H_2_O+H]^+^ | M-Agly-Hex-Hex-4H_2_O |  | |
|  | 163.0607 | C_6_H_11_O_5_+ | 37730 | [M-C_27_H_44_NO_2_-C_5_H_8_O_4_-C_6_H_10_O_5_-C_6_H_10_O_5_+H]^+^ | M-Agly-Pent-Hex-Hex | Hexose fragment | |
|  | 147.0660 | C_6_H_11_O_4_+ | 5620 | [M-C_27_H_44_NO_2_-C_5_H_8_O_5_-C_6_H_10_O_5_-C_6_H_10_O_5_+H]^+^ | M-Agly-Pent-Hex-Hex | Deoxyhexose fragment | |
|  | 145.0500 | C_6_H_9_O_4_+ | 20872 | [M-C_27_H_44_NO_2_-C_5_H_8_O_4_-C_6_H_10_O_5_-C_6_H_10_O_5_-H_2_O+H]^+^ | M-Agly-Pent-Hex-Hex-H_2_O |  | |
|  | 127.0392 | C_6_H_7_O_3_+ | 7066 | [M-C_27_H_44_NO_2_-C_5_H_8_O_4_-C_6_H_10_O_5_-C_6_H_10_O_5_-2H_2_O+H]^+^ | M-Agly-Pent-Hex-Hex-2H_2_O |  | |
|  | 133.0500 | C_5_H_9_O_4_+ | 180699 | [M-C_27_H_44_NO_2_-C_6_H_10_O_4_-C_6_H_10_O_5_-C_6_H_10_O_5_-O+H]^+^ | M-Agly-DeOxHex-Hex-Hex-Ox | Pentose fragment | |
|  |  |  |  |  |  |  | |
| 23, A, 364.4, PRs/ARs | 886.5169 | C_45_H_76_NO_16_+ | 114998 | [M+H]^+^ | M | Putative molecular ion | |
|  | 724.4640 | C_39_H_66_NO_11_+ | 43344 | [M-C_6_H_10_O_5_]^+^ | M-Hex | Diglycosidic steroid | |
|  | 578.4058 | C_33_H_56_NO_7_+ | 698636 | [M-C_6_H_10_O_5_-C_6_H_10_O_4_]^+^ | M-Hex-DeOxHex | Monoglycosidic steroid | |
|  | **416.3522** | C_27_H_46_NO_2_+ | 1772166 | [M-C_6_H_10_O_5_-C_6_H_10_O_4_-C_6_H_10_O_5_]^+^ | M-Hex-DeOxHex-Hex | Aglycone | |
|  | 325.1125 | C_12_H_21_O_10_+ | 58220 | [M-C_27_H_46_NO_2_-C_6_H_10_O_4_+H]^+^ | M-Agly-DeOxHex | Disaccharide (Hex-Hex) | |
|  | 307.1010 | C_12_H_19_O_9_+ | 1154 | [M-C_27_H_46_NO_2_-C_6_H_10_O_4_-H_2_O+H]^+^ | M-Agly-DeOxHex-H_2_O |  | |
|  | 289.0921 | C_12_H_17_O_8_+ | 4220 | [M-C_27_H_46_NO_2_-C_6_H_10_O_4_-2H_2_O+H]^+^ | M-Agly-DeOxHex-2H_2_O |  | |
|  | 253.0718 | C_12_H_13_O_6_+ | 384 | [M-C_27_H_46_NO_2_-C_6_H_10_O_4_-4H_2_O+H]^+^ | M-Agly-DeOxHex-4H_2_O |  | |
|  | 309.1178 | C_12_H_21_O_9_+ | 125982 | [M-C_27_H_46_NO_2_-C_6_H_10_O_5_+H]^+^ | M-Agly-Hex | Disaccharide (DeOxHex-Hex) | |
|  | 291.1068 | C_12_H_19_O_8_+ | 7914 | [M-C_27_H_46_NO_2_-C_6_H_10_O_5_-H_2_O+H]^+^ | M-Agly-Hex-H_2_O |  | |
|  | 273.0963 | C_12_H_17_O_7_+ | 30898 | [M-C_27_H_46_NO_2_-C_6_H_10_O_5_-2H_2_O+H]^+^ | M-Agly-Hex-2H_2_O |  | |
|  | 255.0864 | C_12_H_15_O_6_+ | 17358 | [M-C_27_H_46_NO_2_-C_6_H_10_O_5_-3H_2_O+H]^+^ | M-Agly-Hex-3H_2_O |  | |
|  | 237.0759 | C_12_H_13_O_5_+ | 3908 | [M-C_27_H_46_NO_2_-C_6_H_10_O_5_-4H_2_O+H]^+^ | M-Agly-Hex-4H_2_O |  | |
|  | 163.0607 | C_6_H_11_O_5_+ | 93682 | [M-C_27_H_46_NO_2_-C_6_H_10_O_4_-C_6_H_10_O_5_+H]^+^ | M-Agly-DeOxHex-Hex | Hexose fragment | |
|  | 147.0655 | C_6_H_11_O_4_+ | 167510 | [M-C_27_H_46_NO_2_-C_6_H_10_O_5_-C_6_H_10_O_5_+H]^+^ | M-Agly-Hex-Hex | Deoxyhexose fragment | |
|  | 129.0548 | C_6_H_9_O_3_+ | 84298 | [M-C_27_H_46_NO_2_-C_6_H_10_O_5_-C_6_H_10_O_5_-H_2_O+H]^+^ | M-Agly-Hex-Hex-H_2_O |  | |
|  |  |  |  |  |  |  | |
| 24, B, 365.8, PRs/ARs | 868.5091 | C_45_H_74_NO_15_+ | 3130604 | [M+H]^+^ | M | Putative molecular ion | |
|  | 722.4499 | C_39_H_64_NO_11_+ | 107812 | [M-C_6_H_10_O_4_]^+^ | M-DeOxHex | Diglycosidic steroid | |
|  | 576.3919 | C_33_H_54_NO_7_+ | 1854939 | [M-C_6_H_10_O_4_-C_6_H_10_O_4_]^+^ | M-DeOxHex-DeOxHex | Monoglycosidic steroid | |
|  | **414.3387** | C_27_H_44_NO_2_+ | 308323 | [M-C_6_H_10_O_4_-C_6_H_10_O_4_-C_6_H_10_O_5_]^+^ | M-DeOxHex-DeOxHex-Hex | Aglycone | |
|  | 309.1193 | C_12_H_21_O_9_+ | 9720 | [M-C_27_H_44_NO_2_-C_6_H_10_O_4_+H]^+^ | M-Agly-DeOxHex | Disaccharide (DeOxHex-Hex) | |
|  | 273.0975 | C_12_H_17_O_7_+ | 6545 | [M-C_27_H_44_NO_2_-C_6_H_10_O_4_-2H_2_O+H]^+^ | M-Agly-DeOxHex-2H_2_O |  | |
|  | 255.0875 | C_12_H_15_O_6_+ | 3857 | [M-C_27_H_44_NO_2_-C_6_H_10_O_4_-3H_2_O+H]^+^ | M-Agly-DeOxHex-3H_2_O |  | |
|  | 237.0764 | C_12_H_13_O_5_+ | 1504 | [M-C_27_H_44_NO_2_-C_6_H_10_O_4_-4H_2_O+H]^+^ | M-Agly-DeOxHex-4H_2_O |  | |
|  | 293.1248 | C_12_H_21_O_8_+ | 66633 | [M-C_27_H_44_NO_2_-C_6_H_10_O_5_+H]^+^ | M-Agly-Hex | Disaccharide (DeOxHex-DeOxHex) | |
|  | 275.1137 | C_12_H_19_O_7_+ | 34235 | [M-C_27_H_44_NO_2_-C_6_H_10_O_5_-H_2_O+H]^+^ | M-Agly-Hex-H_2_O |  | |
|  | 257.1032 | C_12_H_17_O_6_+ | 22936 | [M-C_27_H_44_NO_2_-C_6_H_10_O_5_-2H_2_O+H]^+^ | M-Agly-Hex-2H_2_O |  | |
|  | 239.0924 | C_12_H_15_O_5_+ | 32622 | [M-C_27_H_44_NO_2_-C_6_H_10_O_5_-3H_2_O+H]^+^ | M-Agly-Hex-3H_2_O |  | |
|  | 221.0819 | C_12_H_13_O_4_+ | 2240 | [M-C_27_H_44_NO_2_-C_6_H_10_O_5_-4H_2_O+H]^+^ | M-Agly-Hex-4H_2_O |  | |
|  | 147.0662 | C_6_H_11_O_4_+ | 48973 | [M-C_27_H_44_NO_2_-C_6_H_10_O_4_-C_6_H_10_O_5_+H]^+^ | M-Agly-DeOxHex-Hex | Deoxyhexose fragment | |
|  | 129.0558 | C_6_H_9_O_3_+ | 39495 | [M-C_27_H_44_NO_2_-C_6_H_10_O_4_-C_6_H_10_O_5_-H_2_O+H]^+^ | M-Agly-DeOxHex-Hex-H_2_O |  | |
|  | 111.0447 | C_6_H_7_O_2_+ | 13056 | [M-C_27_H_44_NO_2_-C_6_H_10_O_4_-C_6_H_10_O_5_-2H_2_O+H]^+^ | M-Agly-DeOxHex-Hex-2H_2_O |  | |
|  |  |  |  |  |  |  | |
| 25, A, 377, PRs/ARs | 1034.5564 | C_50_H_84_NO_21_+ | 1010 | [M+H]^+^ | M | Putative molecular ion | |
|  | 872.5017 | C_44_H_74_NO_16_+ | 184 | [M-C_6_H_10_O_5_]^+^ | M-Hex | Triglycosidic steroid | |
|  | 710.4464 | C_38_H_64_NO_11_+ | 476 | [M-C_6_H_10_O_5_-C_6_H_10_O_5_]^+^ | M-Hex-Hex | Diglycosidic steroid | |
|  | **578.4061** | C_33_H_56_NO_7_+ | 288954 | [M-C_6_H_10_O_5_-C_6_H_10_O_5_-C_5_H_8_O_4_]^+^ | M-Hex-Hex-Pent | Monoglycosidic steroid | |
|  | 416.3518 | C_27_H_46_NO_2_+ | 139206 | [M-C_6_H_10_O_5_-C_6_H_10_O_5_-C_5_H_8_O_4_-C_6_H_10_O_5_]^+^ | M-Hex | Aglycone fragment | |
|  | 325.1124 | C_12_H_21_O_10_+ | 56602 | [M-C_27_H_46_NO_2_-C_6_H_10_O_5_-C_5_H_8_O_4_+H]^+^ | M-Agly-Hex-Pent | Disaccharide (Hex-Hex) | |
|  | 295.1021 | C_11_H_19_O_9_+ | 112870 | [M-C_27_H_46_NO_2_-C_6_H_10_O_5_-C_6_H_10_O_5_+H]^+^ | M-Agly-Hex-Hex | Disaccharide (Pent-Hex) | |
|  | 163.0602 | C_6_H_11_O_5_+ | 38114 | [M-C_27_H_46_NO_2_-C_5_H_8_O_4_-C_6_H_10_O_5_-C_6_H_10_O_5_+H]^+^ | M-Agly-Pent-Hex-Hex | Hexose fragment | |
|  | 145.0497 | C_6_H_9_O_4_+ | 25242 | [M-C_27_H_46_NO_2_-C_5_H_8_O_4_-C_6_H_10_O_5_-C_6_H_10_O_5_-H_2_O+H]^+^ | M-Agly-Pent-Hex-Hex-H_2_O |  | |
|  | 133.0496 | C_5_H_9_O_4_+ | 19912 | [M-C_27_H_46_NO_2_-C_6_H_10_O_5_-C_6_H_10_O_5_-C_6_H_10_O_5_+H]^+^ | M-Agly-Hex-Hex-Hex | Pentose fragment | |
|  | 127.0389 | C_6_H_7_O_3_+ | 6964 | [M-C_27_H_46_NO_2_-C_5_H_8_O_4_-C_6_H_10_O_5_-C_6_H_10_O_5_-2H_2_O+H]^+^ | M-Agly-Pent-Hex-Hex-2H_2_O |  | |
|  |  |  |  |  |  |  | |
| 26, A, 379.2, PRs/ARs | 902.5124 | C_45_H_76_NO_17_+ | 140468 | [M+H]^+^ | M | Putative molecular ion | |
|  | 740.4590 | C_39_H_66_NO_12_+ | 15850 | [M-C_6_H_10_O_5_]^+^ | M-Hex | Diglycosidic steroid | |
|  | 578.4069 | C_33_H_56_NO_7_+ | 1612500 | [M-C_6_H_10_O_5_-C_6_H_10_O_5_]^+^ | M-Hex-Hex | Monoglycosidic steroid | |
|  | **416.3529** | C_27_H_46_NO_2_+ | 1801298 | [M-C_6_H_10_O_5_-C_6_H_10_O_5_-C_6_H_10_O_5_]^+^ | M-Hex-Hex-Hex | Aglycone | |
|  | 325.1132 | C_12_H_21_O_10_+ | 318136 | [M-C_27_H_46_NO_2_-C_6_H_10_O_5_+H]^+^ | M-Agly-Hex | Disaccharide (Hex-Hex) | |
|  | 307.1021 | C_12_H_19_O_9_+ | 3974 | [M-C_27_H_46_NO_2_-C_6_H_10_O_5_-H_2_O+H]^+^ | M-Agly-Hex-H_2_O |  | |
|  | 289.0919 | C_12_H_17_O_8_+ | 25330 | [M-C_27_H_46_NO_2_-C_6_H_10_O_5_-2H_2_O+H]^+^ | M-Agly-Hex-2H_2_O |  | |
|  | 271.0813 | C_12_H_15_O_7_+ | 4364 | [M-C_27_H_46_NO_2_-C_6_H_10_O_5_-3H_2_O+H]^+^ | M-Agly-Hex-3H_2_O |  | |
|  | 253.0719 | C_12_H_13_O_6_+ | 1704 | [M-C_27_H_46_NO_2_-C_6_H_10_O_5_-4H_2_O+H]^+^ | M-Agly-Hex-4H_2_O |  | |
|  | 163.0609 | C_6_H_11_O_5_+ | 423984 | [M-C_27_H_46_NO_2_-C_6_H_10_O_5_-C_6_H_10_O_5_+H]^+^ | M-Agly-Hex-Hex | Hexose fragment | |
|  | 145.0502 | C_6_H_9_O_4_+ | 212904 | [M-C_27_H_46_NO_2_-C_6_H_10_O_5_-C_6_H_10_O_5_-H_2_O+H]^+^ | M-Agly-Hex-Hex-H_2_O |  | |
|  | 127.0393 | C_6_H_7_O_3_+ | 42458 | [M-C_27_H_46_NO_2_-C_6_H_10_O_5_-C_6_H_10_O_5_-2H_2_O+H]^+^ | M-Agly-Hex-Hex-2H_2_O |  | |
|  | 109.0284 | C_6_H_5_O_2_+ | 4330 | [M-C_27_H_46_NO_2_-C_6_H_10_O_5_-C_6_H_10_O_5_-3H_2_O+H]^+^ | M-Agly-Hex-Hex-3H_2_O |  | |
|  |  |  |  |  |  |  | |
| 27, A, 384, PRs/ARs | 870.5216 | C_45_H_76_NO_15_+ | 543164 | [M+H]^+^ | M | Putative molecular ion | |
|  | 724.4641 | C_39_H_66_NO_11_+ | 157398 | [M-C_6_H_10_O_4_]^+^ | M-DeOxHex | Diglycosidic steroid | |
|  | **578.4061** | C_33_H_56_NO_7_+ | 867142 | [M-C_6_H_10_O_4_-C_6_H_10_O_4_]^+^ | M-DeOxHex-DeOxHex | Monoglycosidic steroid | |
|  | 416.3524 | C_27_H_46_NO_2_+ | 389770 | [M-C_6_H_10_O_4_-C_6_H_10_O_4_-C_6_H_10_O_5_]^+^ | M-DeOxHex-DeOxHex-Hex | Aglycone | |
|  | 309.1173 | C_12_H_21_O_9_+ | 12288 | [M-C_27_H_46_NO_2_-C_6_H_10_O_4_+H]^+^ | M-Agly-DeOxHex | Disaccharide (DeOxHex-Hex) | |
|  | 291.1070 | C_12_H_19_O_8_+ | 668 | [M-C_27_H_46_NO_2_-C_6_H_10_O_4_-H_2_O+H]^+^ | M-Agly-DeOxHex-H_2_O |  | |
|  | 273.0967 | C_12_H_17_O_7_+ | 6604 | [M-C_27_H_46_NO_2_-C_6_H_10_O_4_-2H_2_O+H]^+^ | M-Agly-DeOxHex-2H_2_O |  | |
|  | 255.0852 | C_12_H_15_O_6_+ | 3130 | [M-C_27_H_46_NO_2_-C_6_H_10_O_4_-3H_2_O+H]^+^ | M-Agly-DeOxHex-3H_2_O |  | |
|  | 237.0760 | C_12_H_13_O_5_+ | 1530 | [M-C_27_H_46_NO_2_-C_6_H_10_O_4_-4H_2_O+H]^+^ | M-Agly-DeOxHex-4H_2_O |  | |
|  | 293.1236 | C_12_H_21_O_8_+ | 57150 | [M-C_27_H_46_NO_2_-C_6_H_10_O_5_+H]^+^ | M-Agly-Hex | Disaccharide (DeOxHex-DeOxHex) | |
|  | 275.1122 | C_12_H_19_O_7_+ | 28008 | [M-C_27_H_46_NO_2_-C_6_H_10_O_5_-H_2_O+H]^+^ | M-Agly-Hex-H_2_O |  | |
|  | 257.1020 | C_12_H_17_O_6_+ | 18054 | [M-C_27_H_46_NO_2_-C_6_H_10_O_5_-2H_2_O+H]^+^ | M-Agly-Hex-2H_2_O |  | |
|  | 239.0914 | C_12_H_15_O_5_+ | 26654 | [M-C_27_H_46_NO_2_-C_6_H_10_O_5_-3H_2_O+H]^+^ | M-Agly-Hex-3H_2_O |  | |
|  | 221.0815 | C_12_H_13_O_4_+ | 1546 | [M-C_27_H_46_NO_2_-C_6_H_10_O_5_-4H_2_O+H]^+^ | M-Agly-Hex-4H_2_O |  | |
|  | 147.0655 | C_6_H_11_O_4_+ | 43280 | [M-C_27_H_46_NO_2_-C_6_H_10_O_4_-C_6_H_10_O_5_+H]^+^ | M-Agly-DeOxHex-Hex | Deoxyhexose fragment | |
|  | 129.0549 | C_6_H_9_O_3_+ | 30492 | [M-C_27_H_46_NO_2_-C_6_H_10_O_4_-C_6_H_10_O_4_-H_2_O+H]^+^ | M-Agly-DeOxHex-DeOxHex-H_2_O |  | |
|  | 111.0440 | C_6_H_7_O_2_+ | 9344 | [M-C_27_H_46_NO_2_-C_6_H_10_O_4_-C_6_H_10_O_4_-2H_2_O+H]^+^ | M-Agly-DeOxHex-DeOxHex-2H_2_O |  | |
|  |  |  |  |  |  |  | |
| 28, B, 396.4, PRs/ARs | 954.5072 | C_48_H_76_NO_18_+ | 62459 | [M+H]^+^ | M | Putative molecular ion | |
|  | 808.4477 | C_42_H_66_NO_14_+ | 5724 | [M-C_6_H_10_O_4_]^+^ | M-DeOxHex | Diglycosidic steroid | |
|  | 662.3920 | C_36_H_56_NO_10_+ | 150900 | [M-C_6_H_10_O_4_-C_6_H_10_O_4_]^+^ | M-DeOxHex-DeOxHex | Malonyl-glycosidic steroid | |
|  | **414.3371** | C_27_H_44_NO_2_+ | 62699 | [M-C_6_H_10_O_4_-C_6_H_10_O_4_-C_9_H_12_O_8_]^+^ | M-DeOxHex-DeOxHex-MalonylGluc | Aglycone | |
|  | 293.1236 | C_12_H_21_O_8_+ | 21563 | [M-C_27_H_44_NO_2_-C_9_H_12_O_8_+H]^+^ | M-Agly-MalonylHex | Disaccharide (DeOxHex-DeOxHex) | |
|  | 163.0607 | C_6_H_11_O_5_+ | 7362 | [M-C_27_H_44_NO_2_-C_6_H_10_O_4_-C_6_H_10_O_4_-C_3_H_2_O_3_+H]^+^ | M-Agly-DeOxHex-DeOxHex-Malonyl group | Hexose fragment | |
|  | 147.0658 | C_6_H_11_O_4_+ | 19564 | [M-C_27_H_44_NO_2_-C_6_H_10_O_4_-C_9_H_12_O_8_+H]^+^ | M-Agly-DeOxHex-MalonylHex | Deoxyhexose fragment | |
|  | 129.0553 | C_6_H_9_O_3_+ | 14877 | [M-C_27_H_44_NO_2_-C_6_H_10_O_4_-C_9_H_12_O_8_-H_2_O+H]^+^ | M-Agly-DeOxHex-MalonylHex-H_2_O |  | |
|  | 145.0499 | C_6_H_9_O_4_+ | 4435 | [M-C_27_H_44_NO_2_-C_6_H_10_O_4_-C_6_H_10_O_4_-C_3_H_2_O_3_-H_2_O+H]^+^ | M-Agly-DeOxHex-DeOxHex-Malonyl group-2H_2_O |  |  |
|  | 127.0392 | C_6_H_7_O_3_+ | 3095 | [M-C_27_H_44_NO_2_-C_6_H_10_O_4_-C_6_H_10_O_4_-C_3_H_2_O_3_-2H_2_O+H]^+^ | M-Agly-DeOxHex-DeOxHex-Malonyl group-H_2_O |  |  |
|  | 249.0602 | C_9_H_13_O_8_+ | 1053 | [M-C_27_H_44_NO_2_-C_6_H_10_O_4_-C_6_H_10_O_4_+H]^+^ | M-Agly-DeOxHex-DeOxHex | Malonyl-hexose fragment | |
|  |  |  |  |  |  |  | |
| 29, G, 396.4, PRs/ARs | **741.4440** | C_39_H_65_O_13_+ | 26805 | [M+H]^+^ | M | Putative molecular ion | |
|  | 579.3902 | C_33_H_55_O_8_+ | 18236 | [M-C_6_H_10_O_5_]^+^ | M-Hex | Monoglycosidic steroid | |
|  | 417.3398 | C_27_H_45_O_3_+ | 1020 | [M-C_6_H_10_O_5_-C_6_H_10_O_5_]^+^ | M-Hex-Hex | Aglycone | |
|  |  |  |  |  |  |  | |
| 30, G, 400.1, PRs/ARs | 1045.5259 | C_51_H_81_O_22_+ | 101 | [M+H]^+^ | M | Putative molecular ion | |
|  | 883.4692 | C_45_H_71_O_17_+ | 994 | [M-C_6_H_10_O_5_]^+^ | M-Hex | Triglycosidic steroid | |
|  | 737.4143 | C_39_H_61_O_13_+ | 552 | [M-C_6_H_10_O_5_-C_6_H_10_O_4_]^+^ | M-Hex-DeOxHex | Diglycosidic steroid | |
|  | 575.3599 | C_33_H_51_O_8_+ | 11405 | [M-C_6_H_10_O_5_-C_6_H_10_O_4_-C_6_H_10_O_5_]^+^ | M-Hex-DeOxHex-Hex | Monoglycosidic steroid | |
|  | **413.3062** | C_27_H_41_O_3_+ | 28574 | [M-C_6_H_10_O_5_-C_6_H_10_O_4_-C_6_H_10_O_5_-C_6_H_10_O_5_]^+^ | M-Hex-DeOxHex-Hex-Hex | Aglycone | |
|  | 325.1132 | C_12_H_21_O_10_+ | 525 | [M-C_27_H_41_O_3_-C_6_H_10_O_4_-C_6_H_10_O_5_+H]^+^ | M-Agly-DeOxHex-Hex | Disaccharide (Hex-Hex) | |
|  | 307.1042 | C_12_H_19_O_9_+ | 182 | [M-C_27_H_41_O_3_-C_6_H_10_O_4_-C_6_H_10_O_5_-H_2_O+H]^+^ | M-Agly-DeOxHex-Hex-H_2_O |  | |
|  | 289.0933 | C_12_H_17_O_8_+ | 336 | [M-C_27_H_41_O_3_-C_6_H_10_O_4_-C_6_H_10_O_5_-2H_2_O+H]^+^ | M-Agly-DeOxHex-Hex-2H_2_O |  | |
|  | 309.1184 | C_12_H_21_O_9_+ | 2672 | [M-C_27_H_41_O_3_-C_6_H_10_O_5_-C_6_H_10_O_5_+H]^+^ | M-Agly-Hex-Hex | Disaccharide (DeOxHex-Hex) | |
|  | 291.1076 | C_12_H_19_O_8_+ | 190 | [M-C_27_H_41_O_3_-C_6_H_10_O_5_-C_6_H_10_O_5_-H_2_O+H]^+^ | M-Agly-Hex-Hex-H_2_O |  | |
|  | 273.0978 | C_12_H_17_O_7_+ | 670 | [M-C_27_H_41_O_3_-C_6_H_10_O_5_-C_6_H_10_O_5_-2H_2_O+H]^+^ | M-Agly-Hex-Hex-2H_2_O |  | |
|  | 255.0869 | C_12_H_15_O_6_+ | 637 | [M-C_27_H_41_O_3_-C_6_H_10_O_5_-C_6_H_10_O_5_-3H_2_O+H]^+^ | M-Agly-Hex-Hex-3H_2_O |  | |
|  | 163.0618 | C_6_H_11_O_5_+ | 812 | [M-C_27_H_41_O_3-_C_6_H_10_O_4_-C_6_H_10_O_5_-C_6_H_10_O_5_+H]^+^ | M-Agly-DeOxHex-Hex-Hex | Hexose fragment | |
|  | 147.0660 | C_6_H_11_O_4_+ | 4228 | [M-C_27_H_41_O_3-_C_6_H_10_O_5_-C_6_H_10_O_5_-C_6_H_10_O_5_+H]^+^ | M-Agly-Hex-Hex-Hex | Deoxyhexose fragment | |
|  |  |  |  |  |  |  | |
| 31, G, 409, PRs/ARs | 1047.5410 | C_51_H_83_O_22_+ | 1104 | [M+H]^+^ | M | Putative molecular ion | |
|  | 901.4828 | C_45_H_73_O_18_+ | 115 | [M-C_6_H_10_O_4_]^+^ | M-DeOxHex | Triglycosidic steroid | |
|  | 739.4281 | C_39_H_63_O_13_+ | 5249 | [M-C_6_H_10_O_4_-C_6_H_10_O_5_]^+^ | M-DeOxHex-Hex | Diglycosidic steroid | |
|  | **577.3756** | C_33_H_53_O_8_+ | 23757 | [M-C_6_H_10_O_4_-C_6_H_10_O_5_-C_6_H_10_O_5_]^+^ | M-DeOxHex-Hex-Hex | Monoglycosidic steroid | |
|  | 415.3214 | C_27_H_43_O_3_+ | 3482 | [M-C_6_H_10_O_4_-C_6_H_10_O_5_-C_6_H_10_O_5_-C_6_H_10_O_5_]^+^ | M-DeOxHex-Hex-Hex | Aglycone | |
|  | 325.1120 | C_12_H_21_O_10_+ | 367 | [M-C_27_H_43_O_3_-C_6_H_10_O_4_-C_6_H_10_O_5_+H]^+^ | M-Agly-DeOxHex-Hex | Disaccharide (Hex-Hex) | |
|  | 307.1020 | C_12_H_19_O_9_+ | 68 | [M-C_27_H_43_O_3_-C_6_H_10_O_4_-C_6_H_10_O_5_-H_2_O+H]^+^ | M-Agly-DeOxHex-Hex-H_2_O |  | |
|  | 289.0942 | C_12_H_17_O_8_+ | 146 | [M-C_27_H_43_O_3_-C_6_H_10_O_4_-C_6_H_10_O_5_-2H_2_O+H]^+^ | M-Agly-DeOxHex-Hex-2H_2_O |  | |
|  | 309.1186 | C_12_H_21_O_9_+ | 3029 | [M-C_27_H_43_O_3_-C_6_H_10_O_5_-C_6_H_10_O_5_+H]^+^ | M-Agly-Hex-Hex | Disaccharide (DeOxHex-Hex) | |
|  | 273.0969 | C_12_H_17_O_7_+ | 833 | [M-C_27_H_43_O_3_-C_6_H_10_O_5_-C_6_H_10_O_5_-2H_2_O+H]^+^ | M-Agly-Hex-Hex-2H_2_O |  | |
|  | 255.0871 | C_12_H_15_O_6_+ | 422 | [M-C_27_H_43_O_3_-C_6_H_10_O_5_-C_6_H_10_O_5_-3H_2_O+H]^+^ | M-Agly-Hex-Hex-3H_2_O |  | |
|  | 163.0610 | C_6_H_11_O_5_+ | 1123 | [M-C_27_H_43_O_3-_C_6_H_10_O_4_-C_6_H_10_O_5_-C_6_H_10_O_5_+H]^+^ | M-Agly-DeOxHex-Hex-Hex | Hexose fragment | |
|  | 147.0658 | C_6_H_11_O_4_+ | 5160 | [M-C_27_H_43_O_3-_C_6_H_10_O_5_-C_6_H_10_O_5_-C_6_H_10_O_5_+H]^+^ | M-Agly-Hex-Hex-Hex | Deoxyhexose fragment | |
